# Molecular Mechanisms by Which S100A4 Regulates the Migration and Invasion of PGCCs With Their Daughter Cells in Human Colorectal Cancer

**DOI:** 10.3389/fonc.2020.00182

**Published:** 2020-02-21

**Authors:** Fei Fei, Kai Liu, Chunyuan Li, Jiaxing Du, Zhen Wei, Bo Li, Yuwei Li, Yi Zhang, Shiwu Zhang

**Affiliations:** ^1^Department of Pathology, Tianjin Union Medical Center, Tianjin, China; ^2^Department of Oncology, Nanjing First Hospital, Nanjing Medical University, Nanjing, China; ^3^Graduate School, Tianjin Medical University, Tianjin, China; ^4^Graduate School, Tianjin University of Traditional Chinese Medicine, Tianjin, China; ^5^Departments of Colorectal Surgery, Tianjin Union Medical Center, Tianjin, China

**Keywords:** polyploid giant cancer cells, colorectal cancer, metastasis, S100A4, TRIM21

## Abstract

Recently, an increasing number of evidences have shown that polyploid giant cancer cells (PGCCs) could generate daughter cells with a strong migration and invasion ability, which have been implicated in cancer recurrence and metastasis. However, the underlying molecular mechanisms of PGCCs with their daughter cells remain largely unclear. *In vitro* and *in vivo* experiments combined with 222 cases of human colorectal cancer (CRC) samples were used to identify the molecular mechanisms of S100A4-related proteins regulating the invasion and metastasis of PGCCs with their daughter cells. PGCCs with their daughter cells had high migration, invasion, and proliferation abilities compared to control cells; these were significantly inhibited after S100A4 knockdown. The high expression of cathepsin B, cyclin B1, TRIM21, and Annexin A2 were significantly downregulated after S100A4 knockdown, while the overexpression of S100A4, cathepsin B, cyclin B1, and S100A10 were significantly downregulated after TRIM21 knockdown in PGCCs with their daughter cells. The tumorigenic and metastatic ability of PGCCs with their daughter cells *in vivo* was significantly stronger compared to the untreated cells, which was significantly decreased after S100A4 knockdown. Moreover, the expression of S100A4-related proteins was positively correlated with the malignancy degree of human CRC, and maintained a high level in lymph node metastasis. S100A4 and TRIM21 may regulate each other to affect the expression and subcellular localization of cyclin B1, and participate in regulating the structure and function of Annexin A2/S100A10 complex, affecting downstream cathepsin B, resulting in the invasion and metastasis of PGCCs with their daughter cells. Besides, 14-3-3 ζ/δ and Ezrin may be involved in the motility and invasion of PGCCs with their daughter cells via cytoskeletal constructions with S100A4.

## Introduction

Colorectal cancer (CRC) is the third most commonly diagnosed new cancer (10.2%) and is the second leading cause of death (9.2%) among cancer patients in both sexes worldwide (1). According to the global cancer statistics, over 1.8 million new CRC cases and 880,000 CRC deaths are expected in 2018, which accounts for about 1 in 10 total new cancer cases and deaths (1). Regardless of the remarkable improvement in diagnosis and treatment of CRC, distant metastasis and postoperative relapse remain the leading causes for the unsatisfactory 5-year survival rate and poor prognosis of CRC patients (2, 3).

Recently, numerous reports had showed that cobalt chloride (CoCl_2_) could induce the formation of polyploid giant cancer cells (PGCCs) (4, 5). PGCCs, a special subpopulation of cancer cells, are defined by size and morphology, and not cell surface markers (6, 7), and are considered the seed cells fueling the growth, metastasis, chemoresistance, recurrence, and patients' prognosis in many kinds of human malignant tumors (6–10). PGCCs have many properties of cancer stem cells and contribute to malignant solid tumor heterogeneity (5, 10). The number of PGCCs is more in high-grade malignant tumors than in their low-grade counterparts, more in relapse after chemotherapy than in that before chemotherapy, and more in the metastatic foci than in the primary sites (6, 9). PGCCs and their generating daughter cells via asymmetric cell division acquire a mesenchymal phenotype and express less epithelial markers, which correlates with tumor cell infiltration and invasion (6, 11). However, the underlying molecular mechanisms involved in the invasion and metastasis of PGCCs and their daughter cells remain largely unclear.

We previously used a high-throughput iTRAQ-based proteomic methodology coupled with liquid chromatography-electrospray ionization tandem mass spectroscopy to determine the differentially expressed proteins in the HEY and SKOv3 human ovarian cancer cell lines with and without CoCl_2_ and confirmed that S100A4 was significantly upregulated in PGCCs and their budding daughter cells (11). S100A4, a metastasis-related protein, also named calvasculin, metastasin, p9Ka, 18A2, 42A, CAPL, pEL-98, and fibroblast-specific protein, is a member of S100 calcium-binding protein family (12). The human S100A4 protein is an X-type four-helix bundle symmetrical homomeric dimer, which is coded by the *S100A4* gene (localized in chromosome 1q21) and contains 101 amino acid residues with molecular masses of 10–12 kDa (13). S100A4 was first identified to correlate with cancer metastasis in 1989, followed by the finding that high S100A4 transfection could strengthen the tumorigenic potential and metastatic phenotype *in vivo* (14, 15). S100A4 contributes to the progression and metastasis of numerous cancers via both intracellular and extracellular pathways, which influence the stability of lamellipodia and chemotactic cell migration through the targeting of the intracellular cytoskeleton and extracellularly stimulating angiogenesis, promoting the secretion of various cytokines from cancer cells (16, 17). Here, this study was to investigate the underlying molecular events concerning S100A4 in PGCCs with their daughter cells contributing to the invasion and metastasis of human CRC *in vivo* and *in vitro*, which might lead to a better understanding of CRC progression.

## Materials and Methods

### Cell Lines and Culture

The human CRC cell lines in this study (LoVo and HCT116) were achieved from the American Type Culture Collection. LoVo and HCT116 were both cultured in RPMI-1640 medium supplemented with 10% fetal bovine serum (FBS), 100 U/mL penicillin, and 100 μg/mL streptomycin (Complete medium). The above agents were obtained from Thermo Fisher Scientific. The cells were incubated at 37°C and conditions of 5% CO_2_ under moderate humidity.

### Formation of PGCCs Induced by CoCl_2_ Treatment

Four hundred fifty micrometers CoCl_2_ (Sigma-Aldrich, St. Louis, MO, USA) was added in the medium of LoVo and HCT116 for different durations based on their individual resistance to hypoxia. After CoCl_2_ treatment, most of cells were died while a few giant cells survived. We have described the properties of PGCCs in our previous published papers (5, 18–20). Ten days or so after CoCl_2_ treatment, the surviving PGCCs could generate daughter cells through asymmetric cell division. After treatment with CoCl_2_ 3–4 times, the PGCCs occupied 20–30% total cells and 70–80% were the PGCCs-derived daughter cells. Based on the long-term experimental data and observation, we defined the PGCC as a cancer cell that was at least three times larger in size than that of regular cancer cells. The size of each PGCC nucleus was measured using a micrometer in our study.

### Cell Migration Assay

Cell migratory ability of LoVo and HCT116 was performed by wound-healing assay and transwell migration assay. Cells overgrowed the 6-well plates; then, scratched the monolayer cells by sterile pipette tips uniformly, washed away the cast-off cells with phosphate-buffered saline (PBS), and incubated in serum-free medium. Image-J software was used to measure the scratched area. Cell migratory ability of wound-healing was assessed using the following formula: [(wound area at 0 h) – (wound area at indicated 24 h)] / (wound area at 0 h) (6). A higher score indicates a better migratory ability.

Cell culture inserts (8 μm; Corning Inc.; 24-well plate) were used in transwell migration assay. 10 × 10^4^ cells in 200 μL medium (supplemented with 1% FBS) were seeded in the upper chamber, while 600 μL medium (supplemented with 20% FBS) was added in the lower chamber. After incubating for 24 h, fixed the cell culture inserts with absolute methanol for 30 min and then stained using 0.1% crystal violet for 30 min. Cell migratory ability of transwell migration assay was assessed by the method: counting the average number of stained cells per field (100×, at least five different fields). The more stained cells, the better cell migratory ability.

### Cell Invasion Assay

Cell invasive ability was performed by the transwell invasion assay (8 μm; Corning Inc.). Added the cell suspensions (including 5 × 10^5^ cells in 200 μL medium supplemented with 1% FBS) onto the inserts which were pre-coated with matrigel basement membrane matrix. Then, placed the inserts in the bottom chamber contained 600 μL medium (supplemented with 1% FBS), incubating for 24 h. Fixed the inserts with absolute methanol for 30 min and then stained with 0.1% crystal violet for 30 min. Cell invasive ability of transwell invasion assay was assessed by counting the average number of stained cells per field (100×, at least five different fields). The more stained cells, the better cell invasive ability.

### Plate Colony Formation Assay

Cultured tumor cells in the logarithmic phase and 60, 120, and 240 cells were seeded into 6-well plates, followed by incubation for 2 weeks. When macroscopic cell colonies appeared in the bottom of the plate, the cells were fixed with absolute methanol for 20 min, and then stained the cell colonies with 0.1% crystal violet for 30 min. Counted the number of cell colonies visually using a microscope (clusters containing ≥50 cells were counted as a single colony). The colony formation efficiency was defined as the number of cell colonies/seeded cells.

### Western Blots (WB) Analysis

WB analyses were carried out to detect the expression of molecules related to S100A4 in LoVo and HCT116 cells and nude mice tumor tissues. The total, nuclear, and cytoplasmic protein were extracted by the corresponding manufacturer's instructions, respectively (Thermo Fisher Scientific, Inc.). Detailed WB procedures were performed as described previously (6). Information regarding the antibodies used has been listed in Table S1. And β-actin was indicated as the protein-loading control in this study. Quantitative analysis of the gray value of target protein band was normalized to that of the loading control band. The gray value of each protein band was determined by Image-J. All WB experiments were repeated multiple times.

### Immunocytochemical (ICC) and Immunofluorescence Staining

Monolayer cells were cultured on glass coverslips until they attained 70–80% confluence and were then fixed with 75% ethanol for 30 min. After being washed with PBS thrice, these slides were incubated with endogenous peroxidase inhibitor for 15 min, and blocked with goat serum for 20 min, followed by treatment with different primary antibodies at 4°C overnight (information regarding the antibodies used has been listed in Table S1). The slides were then incubated with biotin-labeled goat anti-mouse/rabbit IgG for 20 min and horseradish-labeled streptomycin for 15 min. Detailed ICC analysis was performed referring to the instructions of Biotin-Streptavidin HRP Detection Systems (SP-9000, Zhongshan Inc.). PBS was used as a negative control to replace the primary antibody. For immunofluorescence staining, the cells were incubated with primary antibodies 4°C overnight (information regarding the antibodies used has been listed in Table S1), followed by reacting with the fluorescein (FITC)-conjugated Affinipure Goat Anti-Rabbit IgG(H+L) (SA00003-2, Proteintech) at room temperature for 30 min. The nuclei were visualized with DAPI. The cell morphology was visualized and photographed with fluorescence microscopy.

### Transient siRNA Transfection

Cells (~40–60% confluence) were transfected for 48–72 h with small interfering RNAs (siRNAs) targeted to the human *S100A4* and *tripartite motif-containing 21 (TRIM21)* genes (sequences of siRNAs have been listed in Table S1) at a 50-nM concentration (pre-experimental conditions have been shown in Figures S1A,B). The siRNA sequences were constructed by Shanghai Genepharma and transfected with the lipofectamine RNAiMax (Thermo) [siRNA: lipo = 20:1 (pmol:ul)].

### Co-immunoprecipitation (Co-IP) and Mass Spectrometry (MS)

Cells were lysed with IP lysis buffer (Thermo) containing 1× Halt Protease & Phosphatase Inhibitor Cocktail for 30 min on ice, followed by centrifugation at 14,000 *g* for 10 min. The samples were then incubated with rabbit anti-S100A4 monoclonal antibodies (IP application, 1:50) at 4°C overnight; normal rabbit IgG (Beyotime, Shanghai, China) was used as the negative control. Next, pre-washed protein A/G agarose beads (Thermo) was added to the mixture and mixed for 2 h at 4°C on a roller. After washing and centrifugation, the immunoprecipitates were examined by silver staining and WB using anti-S100A4 antibodies. MS analysis of coprecipitation substrates was performed using tandem mass spectrometry (MS/MS) in Q ExactiveTM plus (Thermo) coupled online to the ultra-performance liquid chromatography system for the acquisition of MS/MS data. The peptides were identified and quantified using Proteome Discoverer 1.3. The peptide confidence was set at high, and peptide ion score was set at a value >20.

### Animal Experiments

Fifty-five BALB/cNU/NU nude mice (7 weeks old) were obtained from Beijing Weitonglihua Co. Ltd. Thirty nude mice injected with LoVo and 25 nude mice injected HCT116, which were both divided into three groups, including control cells without CoCl_2_ treatment (Control), PGCCs with their daughter cells (Treatment), PGCCs with their daughter cells after S100A4 knockdown (Si-Treatment). 1 × 10^6^ cells were resuspended in 200 μL of PBS and injected in the right flank of each mouse. Starting on the 11th day after LoVo cell inoculation and 7th day after HCT116 cell inoculation, tumors were visible and measured every other day. The tumor volume (mm^3^) = (length × width^2^)/2 (21). On the 37th day and 19th day after inoculation, the LoVo cell-injected and HCT116 cell-injected mice, respectively, were sacrificed, and the tumor, liver, and lung tissues were harvested. The animal study was approved and supported by the Institutional Animal Care and Use Committee of Tianjin Union Medicine Center.

### Hematoxylin & Eosin (H&E) Staining

Four micrometers-thick sections of the paraffin-embedded tissues were subjected to deparaffinization, rehydrated, and counterstained with hematoxylin and eosin for 1 min; then dehydrated, made transparent, and mounted onto coverslips.

### Human Tissues Samples

All the human CRC paraffin-embedded tissue samples (*n* = 222) were obtained from 2009 to 2013 in the Department of Pathology of Tianjin Union Medical Center. All cases were histologically diagnosed, and none received treatment prior to surgical resection of the tumor. The cases were divided into four groups: 51 well-differentiated CRC primary focus (group I), 55 moderately differentiated CRC primary focus (group II), 52 poorly differentiated CRC primary focus (group III), and 64 lymph node metastasis (group IV). The use of these human tissue samples was approved and supported by the Hospital Review Board of Tianjin Union Medicine Center. All patient information will be kept strictly confidential.

### Immunohistochemical (IHC) Staining and Quantification

IHC staining was performed according to the protocol of Biotin-Streptavidin HRP Detection Systems (SP-9000, Zhongshan Inc.); the detailed information of antibodies was listed in Table S1 and detailed process has been described previously (6). The sum of the positive cell ratio score and staining intensity score was defined as the final score (staining index) of each case. The positive cell ratio score was defined as follows: 0, <5%; 1, ≥5% and <30%; 2, ≥30% and <50%; 3, ≥50%. The staining intensity score was defined as follows: 0, no staining (negative); 1, faint yellow (weak); 2, brownish-yellow (moderate); 3, brown (strong).

### Statistical Analysis

SPSS 17.0 statistical software (IBM Corporation, Armonk, NY) was utilized to analyze all data in this study. The comparisons of tumor growth in the inoculated nude mice and protein expression levels in human CRC tissues were both analyzed by the Kruskal-Wallis test. The comparisons between two groups in tumor growth and in protein expression levels were both analyzed by the Mann-Whitney test. The Pearson's chi-square (χ^2^) test was performed to compare the tumor formation rate and metastasis rate, and some comparisons were analyzed by the two-tailed Student's *t*-test. Data of tumor growth in nude mice and protein expression in human tissues and all histogram data were expressed as mean ± SD. ^*^P < 0.05 was considered statistically significant.

## Results

### Change of Migration, Invasion, Proliferation and the Expression of S100A4, Cathepsin B, and Cyclin B1 After CoCl_2_ Treatment

**Figure 1:**
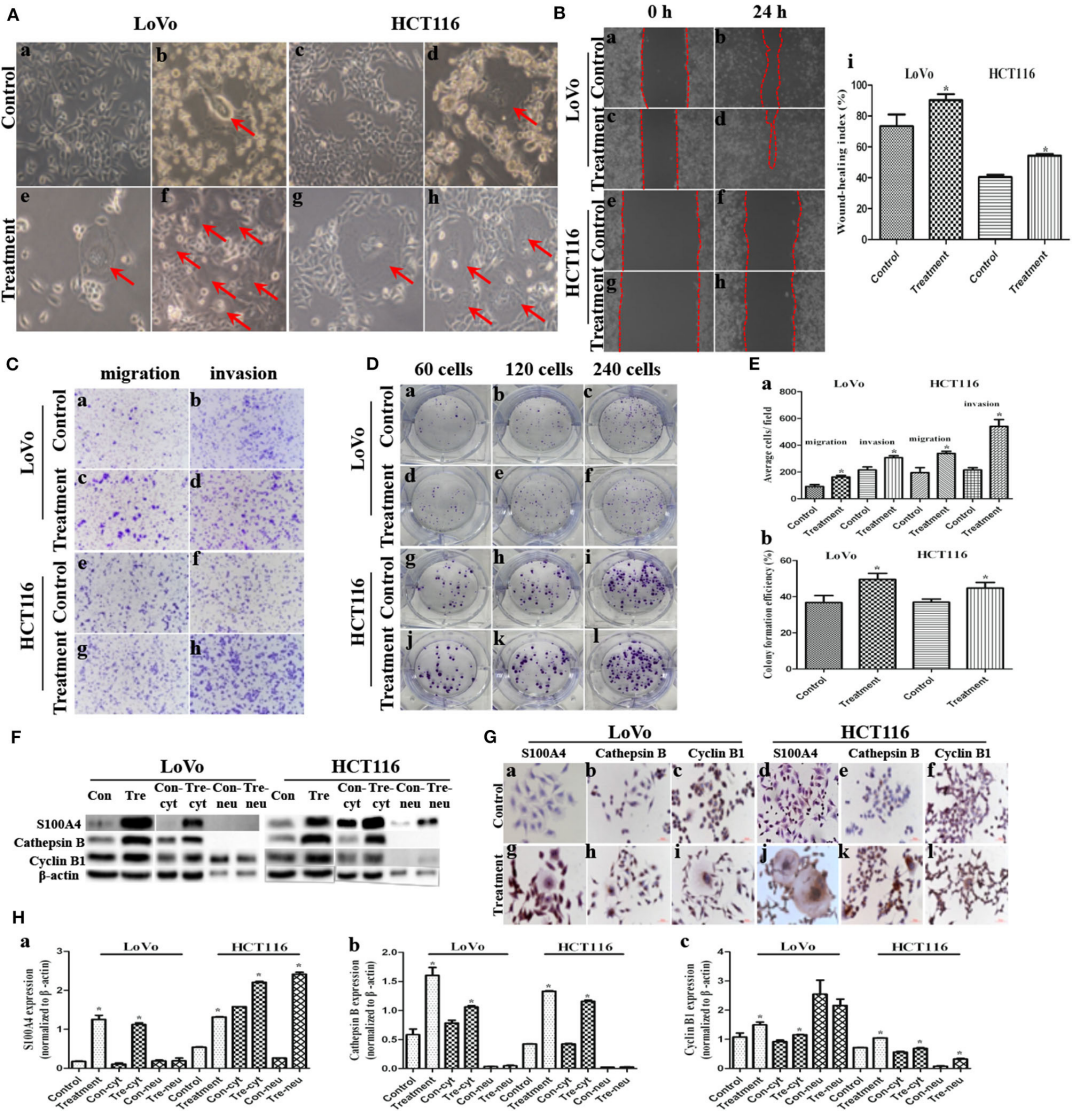
Migration, invasion, proliferation, and expression of S100A4, cathepsin B, and cyclin B1 after CoCl_2_ (^*^P < 0.05). **(A)** Morphologic features before and after CoCl_2_ (100×). (a) LoVo and (c) HCT116 control cells. (b) LoVo and (d) HCT116 after treatment for 48-72 h, red arrows indicate the PGCCs. (e) LoVo and (g) HCT116 PGCCs generating daughter cells via budding after the recovery, red arrows indicate the PGCCs. (f) LoVo and (h) HCT116 show more PGCCs after treatment for 3–4 times, red arrows indicate the PGCCs. **(B)** Wound-healing before and after treatment (100×). LoVo control cells at 0 h (a) and 24 h (b). LoVo after treatment at 0 h (c) and 24 h (d). HCT116 control cells at 0 h (e) and 24 h (f). HCT116 after treatment at 0 h (g) and 24 h (h). (i) Wound-healing index before and after treatment. **(C)** Transwell assay before and after CoCl_2_ (100×). (a) Migration and (b) invasion of LoVo control cells. (c) Migration and (d) invasion of LoVo cells after treatment. (e) Migration and (f) invasion of HCT116 control cells. (g) Migration and (h) invasion of HCT116 cells after treatment. **(D)** Colony formation before and after CoCl_2_. (a) 60, (b) 120, and (c) 240 LoVo control cells. (d) 60, (e) 120, and (f) 240 LoVo cells after treatment. (g) 60, (h) 120, and (i) 240 HCT116 control cells. (j) 60, (k) 120, and (l) 240 HCT116 cells after treatment. **(E)** (a) Cell migration and invasion before and after treatment. (b) Colony formation efficiency before and after treatment. **(F)** The total, cytoplasmic (con-cyt and tre-cyt), and nuclear (con-neu and tre-neu) expression of S100A4, cathepsin B, and cyclin B1 before and after treatment. **(G)** ICC staining of S100A4, cathepsin B, and cyclin B1 before and after treatment (400×). (a) S100A4 (b) cathepsin B (c) cyclin B1 in control LoVo cells. (d) S100A4 (e) cathepsin B (f) cyclin B1 in control HCT116 cells. (g) S100A4 (h) cathepsin B (i) cyclin B1 in LoVo cells after treatment. (j) S100A4 (k) cathepsin B (l) cyclin B1 in HCT116 cells after treatment. **(H)** Comparison of S100A4 (a), cathepsin B (b), and cyclin B1 (c).

LoVo and HCT116 were cultured in complete medium (Figures 1Aa,c). On treating cells with 450 μM CoCl_2_ for 48–72 h, almost all the tumor cells were eliminated except few survived PGCCs (Figures 1Ab,d). The nature of PGCCs had been confirmed in our previous researches, exhibiting multinucleated giant cells and mononuclear giant cells. Approximately 15 day after treatment, several daughter cells were generated by PGCCs via budding (Figures 1Ae,g). When PGCCs and their daughter cells approached 80% confluence, treatment with the same CoCl_2_ concentration and incubated time was re-administered. After CoCl_2_ treatment for 3–4 times, more PGCCs were observed among LoVo cells (Figure 1Af) and HCT116 cells (Figure 1Ah). More PGCCs generating more daughter cells that attained a confluence of 70–80% were used for later analyses, which was claimed to be the group of treatment.

The wound spaces between the red dashed lines in LoVo (Figures 1Ba,c) and HCT116 cells (Figures 1Be,g) before and after treatment at 0 h was narrowed at 24 h (Figures 1Bb,d,f,h), which showed a significant increase in migration of PGCCs with their daughter cells among LoVo and HCT116 (Figure 1Bi) cells. Moreover, transwell assay indicated that cells after treatment had stronger migratory (Figures 1Cc,g,Ea) and invasive (Figures 1Cd,h,Ea) abilities than control cells (Figures 1Ca,e,b,f,Ea). The Plate colony formation assay was carried out to detect the proliferative ability of LoVo and HCT116 cells before (Figures 1Da,c,g,i) and after treatment (Figures 1D,d,f,j,l), indicating a higher proliferation capacity in cells after treatment than control cells (Figure 1Eb).

The expression of metastasis-related protein S100A4 was higher in cells after treatment than before treatment, whose upregulation occurred primarily in the cytoplasm and partly in the nucleus of daughter cells (Figures 1F,Ga,g,d,j,Ha). Cathepsin B was associated with cell motility via disruption of the extracellular matrix, whose expression was also higher in cells after treatment than in control cells, and this upregulation occurred completely in the cytoplasm (Figures 1F,Gb,h,e,k,Hb). Cyclin B1 localized in cytoplasm during the interphase and translocated to the nucleus, thereby triggering mitosis (22), which was upregulated in cells after treatment compared to control cells (Figures 1F,Gc,i,f,l,Hc, 2a–d). In PGCCs, cyclin B1 was primarily localized in the cytoplasm involved in the formation of PGCCs via G2/M phase arrest, and then PGCCs generated daughter cells positively expressing cyclin B1 in the nucleus, correlated to the high proliferation activity (Figures 1Gi,l, 2b,d). Thus, the increase of S100A4, cathepsin B, and cyclin B1 might together contribute to robust migration, invasion, and proliferative abilities of PGCCs with their daughter cells, in which the nuclear translocation of S100A4 and cyclin B1 in daughter cells was also involved.

**Figure 2:**
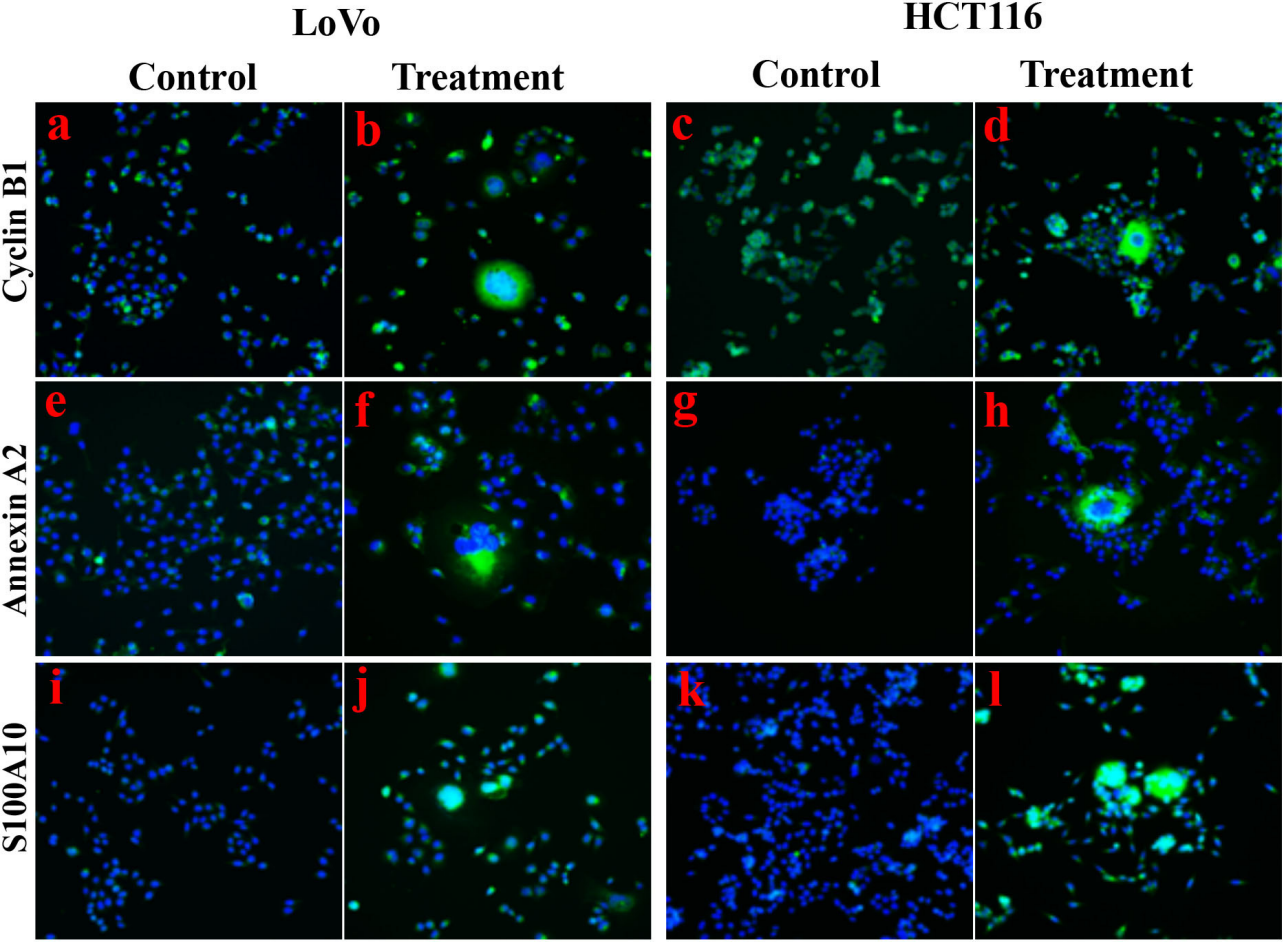
Immunofluorescence staining showed the expression levels and cellular localization of cyclin B1, Annexin A2, and S100A10 before and after CoCl_2_ treatment (400×). **(a)** Cyclin B1 in LoVo control cells. **(b)** Cyclin B1 in LoVo cells after treatment. **(c)** Cyclin B1 in HCT116 control cells. **(d)** Cyclin B1 in HCT116 cells after treatment. **(e)** Annexin A2 in LoVo control cells. **(f)** Annexin A2 in LoVo cells after treatment. **(g)** Annexin A2 in HCT116 control cells. **(h)** Annexin A2 in HCT116 cells after treatment. **(i)** S100A10 in LoVo control cells. **(j)** S100A10 in LoVo cells after treatment. **(k)** S100A10 in HCT116 control cells. **(l)** S100A10 in HCT116 cells after treatment.

### S100A4 Affects Cell Function Cooperating With S100A4-Related Proteins in PGCCs and Daughter Cells

Four siRNAs (116/153/240/358) targeting different sites of human *S100A4*, an siRNA targeting *GAPDH* as the positive control (PC), an unrelated homologous sequence as the negative control (NC), and the mere lipofectamine RNAiMax as the mock control (MC) were transfected in LoVo and HCT116 PGCCs and daughter cells. siRNA-116/153/240/358 all had good inhibiting efficiency in LoVo (Figures S1CDa,Ea) and HCT116 (Figures S1Ca,D,Eb) cells after treatment than with siRNA-PC/NC/MC. Compared with siRNA-PC, WB revealed a robust interfering efficiency of GAPDH in LoVo and HCT116 cells after treatment (Figures S1Cb,D). Here, we chosen siRNA-153 as S100A4 knockdown (si-S100A4) and siRNA-NC as a negative control of S100A4 knockdown (si-Control) for later analysis.

**Figure 3:**
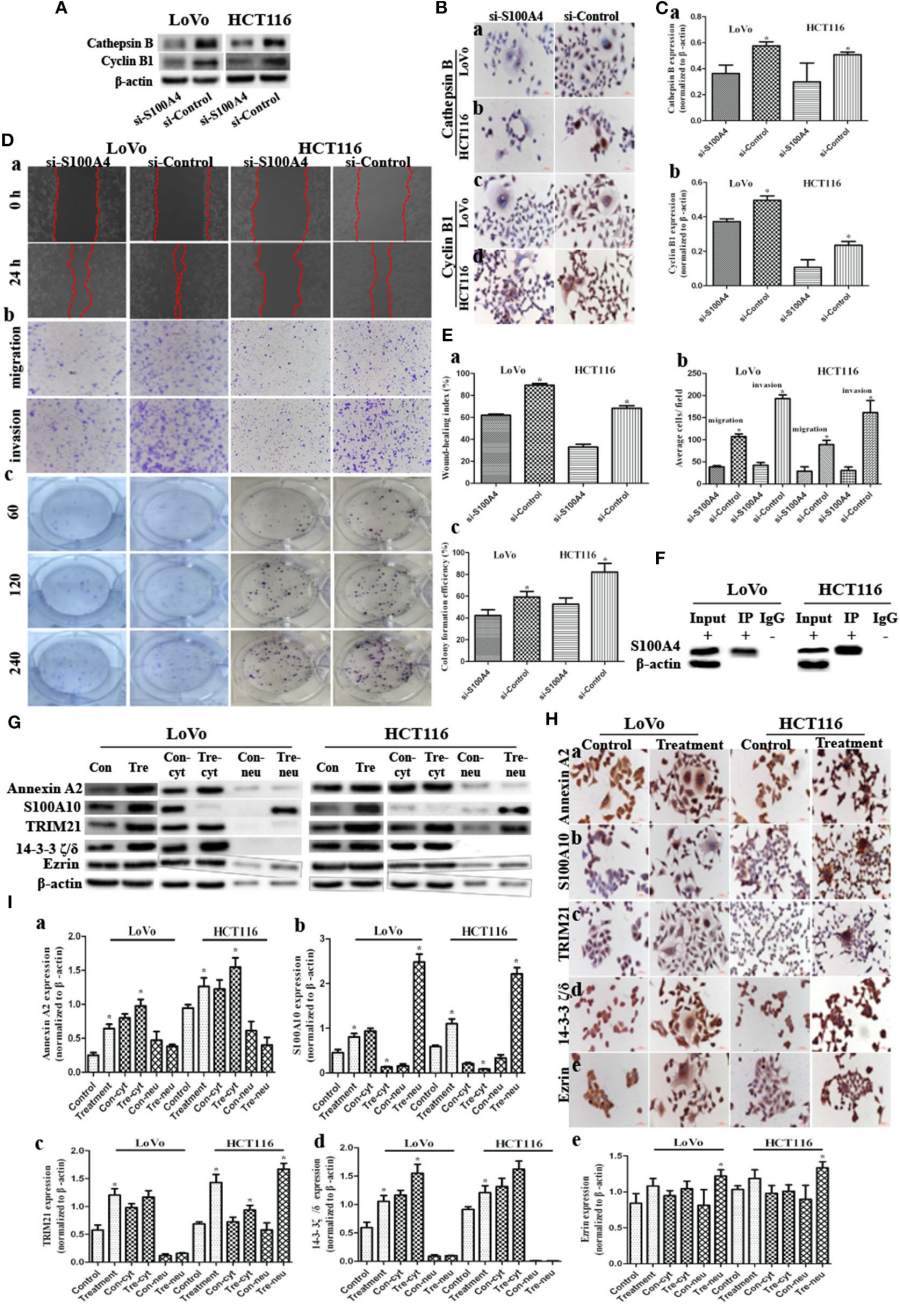
S100A4 affects cell function cooperating with S100A4-related proteins in PGCCs with their daughter cells (^*^P < 0.05). **(A)** WB showed the expression of cathepsin B and cyclin B1 before and after S100A4 knockdown. **(B)** ICC staining of cathepsin B and cyclin B1 before and after S100A4 knockdown. Cathepsin B in (a) LoVo and (b) HCT116. cyclin B1 in (c) LoVo and (d) HCT116. **(C)** Bar graph of WB band intensities for cathepsin B (a) and cyclin B1 (b) before and after S100A4 knockdown. **(D)** S100A4 knockdown of PGCCs and their daughter cells inhibits the migration, invasion, and proliferation. (a) Cell migration in si-S100A4 and si-Control of LoVo (front part) and HCT116 cells (latter part) using wound-healing assay (100×). (b) Cell migration (upper panel) and invasion (lower panel) in si-S100A4 and si-Control of LoVo (front part) and HCT116 (latter part) using transwell assay (100×). (c) Cell proliferation in si-S100A4 and si-Control of LoVo (front part) and HCT116 (latter part) using plate colony formation assay. **(E)** Bar graph of (a) wound-healing, (b) transwell, and (c) plate colony formation before and after S100A4 knockdown. **(F)** Western blots confirmed the results of S100A4 co-immunoprecipitation in LoVo and HCT116 cells after treatment. **(G)** Western blots detected the total, cytoplasmic, and nuclear protein expression differences of Annexin A2, S100A10, TRIM21, 14-3-3 ζ/δ, and Ezrin in LoVo and HCT116 cells before and after treatment (β-actin of Figure 1F was re-used here). **(H)** ICC staining of S100A4-related proteins in LoVo and HCT116 cells before and after treatment (400×). (a) Annexin A2, (b) S100A10, (c) TRIM21, (d) 14-3-3 ζ/δ, (e) Ezrin in LoVo (front part), and HCT116 (latter part). **(I)** Bar graph of WB band intensities for the total, cytoplasmic, and nuclear protein expression differences of Annexin A2 (a), S100A10 (b), TRIM21 (c), 14-3-3 ζ/δ (d), and Ezrin (e) in LoVo and HCT116 cells before and after treatment.

Cathepsin B and cyclin B1 were both downregulated in si-S100A4 compared to si-Control, and the downregulation of cyclin B1 was primarily in the nucleus of daughter cells (Figures 3A–C). The wound-healing assay revealed that healing speed of si-S100A4 in cells after treatment was lower than that in si-Control at 24 h after scratching (Figures 3Da,Ea). Similarly, the transwell assay confirmed that migrated and invaded cells of si-S100A4 in cells after treatment were significantly less than those of si-Control (Figures 3Db,Eb). The plate colony formation assay indicated a subdued proliferation ability in si-S100A4 compared to si-Control (Figures 3Dc,Ec). All data above indicate that S100A4 may inhibit the migration and invasion of PGCCs with daughter cells accompanied by the decrease of cathepsin B and cyclin B1 (a significant decrease in the nuclear translocation of cyclin B1 in daughter cells).

The positive IP results of S100A4 in PGCCs with daughter cells were showed in Figure 3F. Based on the MS analysis (Table S2), we detected that the expression levels of Annexin A2 (Figures 2e–h, 3G,Ha,Ia), S100A10 (Figures 2i–l, 3G,Hb,Ib), TRIM21 (Figures 3G,Hc,Ic), 14-3-3 ζ/δ (Figures 3G,Hd,Id), and Ezrin (Figures 3G,He,Ie) were markedly lower in control cells than in PGCCs with their daughter cells; these upregulations occurring primarily in the cytoplasm, except for S100A10 and Ezrin being upregulated in nucleus of daughter cells, suggests that Annexin A2, S100A10, TRIM21, 14-3-3 ζ/δ, and Ezrin may interact with S100A4 contributing to the migration and invasion of PGCCs with their daughter cells.

### Influence on the S100A4-Related Proteins After S100A4 or TRIM21 Knockdown

**Figure 4:**
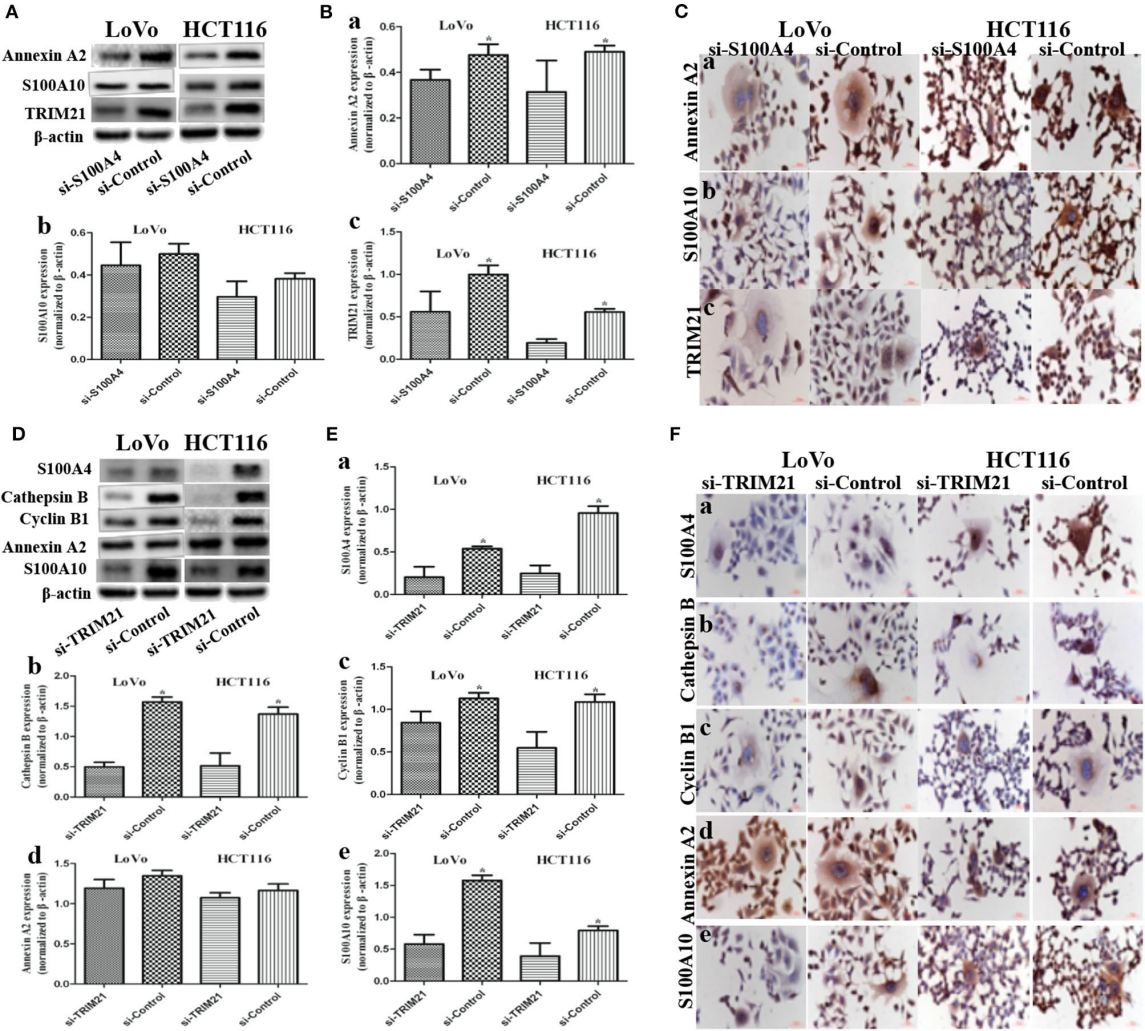
Influence on S100A4-related proteins after S100A4 or TRIM21 knockdown in PGCCs with daughter cells (^*^P < 0.05). **(A)** WB showed the expression of Annexin A2, S100A10, TRIM21 before and after S100A4 knockdown (β-actin of Figure 2A was re-used here and was re-used in Figure S1F). **(B)** Bar graph of WB band intensities for Annexin A2 (a), S100A10 (b), TRIM21 (c) before and after S100A4 knockdown. **(C)** ICC staining of Annexin A2 (a), S100A10 (b), TRIM21 (c) before and after S100A4 knockdown. **(D)** WB showed the expression of S100A4, cathepsin B, cyclin B1, Annexin A2, S100A10 before and after TRIM21 knockdown (β-actin was re-used in Figure S2D). **(E)** Bar graph of WB band intensities for S100A4 (a), cathepsin B (b), cyclin B1 (c), Annexin A2 (d), S100A10 (e) before and after TRIM21 knockdown. **(F)** ICC staining of S100A4 (a), cathepsin B (b), cyclin B1 (c), Annexin A2 (d), S100A10 (e) before and after TRIM21 knockdown.

Annexin A2 (Figures 4A,Ba,Ca) and TRIM21 (Figures 4A,Bc,Cc) were downregulated in si-S100A4 compared to si-Control, and the downregulation occurred primarily in the cytoplasm. There were no obvious differences in S100A10 (Figures 4A,Bb,Cb), 14-3-3 ζ/δ (Figures S1F,Ga,Ha), Ezrin (Figures S1F, Gb,Hb) expression between si-S100A4 and si-Control, but the nuclear translocation of S100A10 in daughter cells was significantly reduced in si-S100A4.

TRIM21, as an E3 ubiquitin protein ligase, was detected in S100A4 interacting proteins, speculated to be directly regulating the ubiquitination of target protein S100A4, followed by influencing degradation or localization or other functions of S100A4. However, we confirmed that S100A4 was not regulated via ubiquitination based on the analysis of S100A4 modification sites in PGCCs with their daughter cells (Table S2). Followed by, three siRNAs (122/404/891) targeting different sites of human *TRIM21*, one siRNA targeting *GAPDH* in PC, one unrelated homologous sequence in NC were transfected in PGCCs with daughter cells. Based on successful inhibition of TRIM21, we chosen siRNA-404 as TRIM21 knockdown (si-TRIM21) and siRNA-NC as a negative control of TRIM21 knockdown (si-Control) for later analysis (Figures S2A–C). S100A4 (Figures 4D,Ea,Fa), cathepsin B (Figures 4D,Eb,Fb), cyclin B1 (Figures 4D,Ec,Fc), and S100A10 (Figures 4D,Ee,Fe) were drastically downregulated in si-TRIM21 cells compared to those in si-Control. However, si-TRIM21 did not influence the expression of Annexin A2 (Figures 4D,Ed,Fd), 14-3-3 ζ/δ (Figures S2D,Ea,Fa), and Ezrin (Figures S2D,Eb,Fb). The downregulation of cathepsin B mainly located in the cytoplasm, while the decrease of S100A4, cyclin B1, and S100A10 were primarily in the nucleus of daughter cells and partly located in PGCCs cytoplasm. These results suggest that TRIM21 may indirectly regulate S100A4 by affecting the expression and subcellular localization of cathepsin B, cyclin B1, and S100A10.

### S100A4-Related Proteins Positively Correlate With Tumorigenesis *in vivo* and the Malignancy of Human CRCs

**Figure 5:**
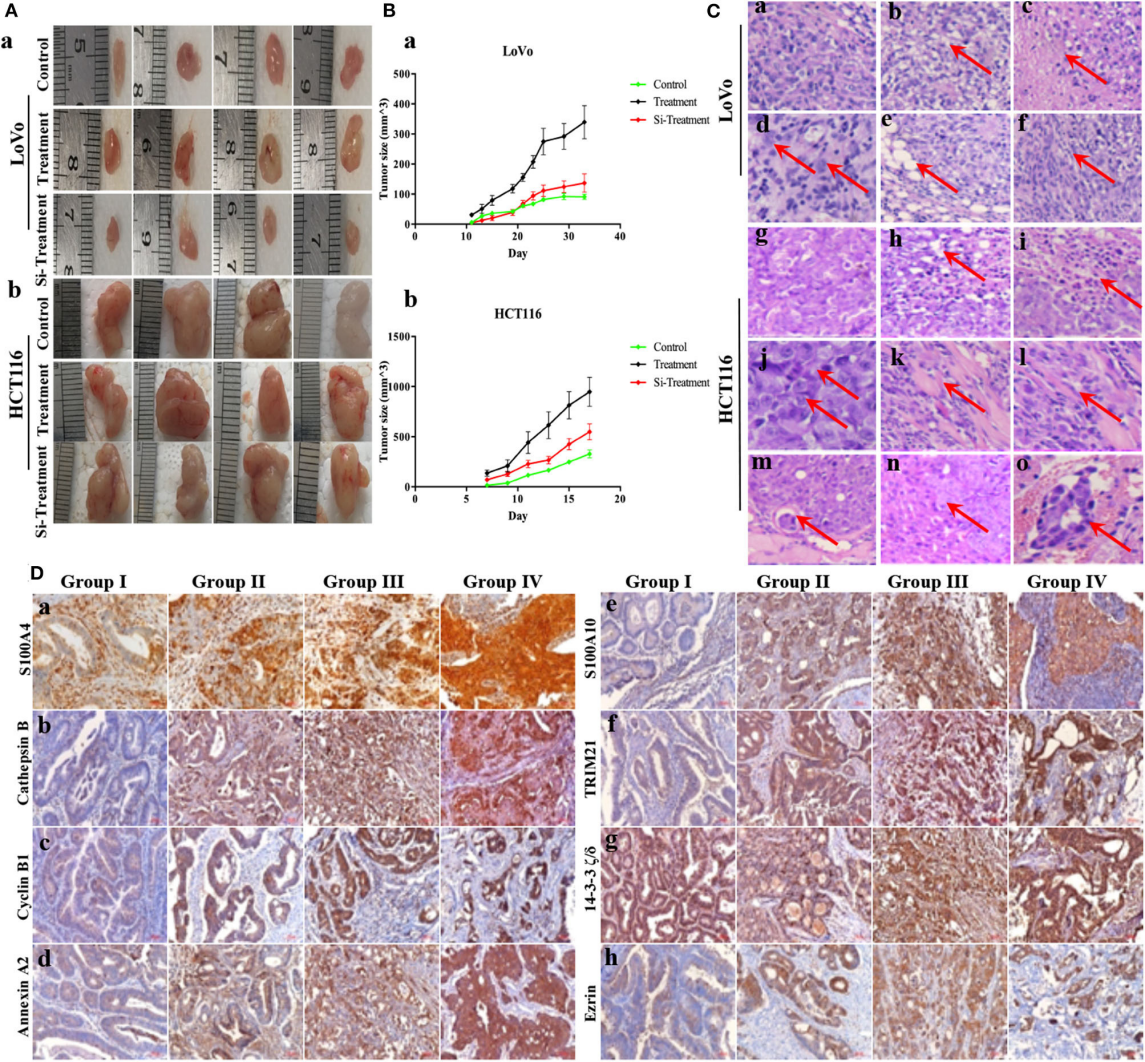
S100A4-related proteins positively correlate with tumorigenesis *in vivo* and the malignancy of human CRCs. **(A)** Characteristics of inoculated tumors with control, treatment, and si-treatment cells. Nude mice inoculated with control, treatment, and si-treatment cells of LoVo (a) and HCT116 (b). **(B)** Tumor growth curves of control, treatment, and si-treatment cells inoculated LoVo (a) and HCT116 (b). **(C)** H&E staining in control, treatment, and si-treatment inoculated cells (200×). (a) LoVo (g) HCT116 control cells. (b) LoVo (h) HCT116 si-treatment and hyaline degeneration (red arrows). (c) LoVo (i) HCT116 si-treatment and necrosis (red arrows). (d) LoVo (j) HCT116 treatment and PGCCs (red arrows) (400×). (e) LoVo treatment and fatty infiltration (k) HCT116 treatment and muscle infiltration (red arrows). (f) LoVo (l) HCT116 treatment and bidirectional differentiation (red arrows). (m) HCT116 treatment and tumor emboli (red arrows). (n) HCT116 treatment and liver metastasis (red arrows). (o) HCT116 treatment and lung metastasis (red arrows). **(D)** IHC staining of S100A4-related proteins in human CRC tissues (200×). (a) S100A4, (b) Cathepsin B, (c) cyclin B1, (d) Annexin A2, (e) S100A10, (f) TRIM21, (g) 14-3-3 ζ/δ, (h) Ezrin in well-differentiated CRC primary focus (group I), moderately differentiated CRC (group II), poorly differentiated CRC (group III), and lymph node metastasis (group IV).

Compared to untreated control cells, treatment (PGCCs with their daughter cells) had higher tumorigenicity and metastasis, evident from the tumor growth speed, size and the time of tumorigenesis (Figures 5A,B and Table 1), while these high tumorigenic and metastatic abilities significantly declined in the group of si-treatment (PGCCs with their daughter cells after S100A4 knockdown) (Figures 5A,B and Table 1). H&E staining was performed to assess tumor morphology and metastasis. Histological examination revealed that the resulting tumor cells of control had a higher nucleus-to-cytoplasm ratio with no necrosis (Figures 5Ca,g). In the si-treatment group, hyaline degeneration occurred in most tumor cells (Figures 5Cb,h) and necrosis (Figures 5Cc,i) was observed. In the treatment group, many PGCCs were observed in the tumor tissue, which is a common histological feature of high-grade malignant tumors (Figures 5Cd,j). Furthermore, other invasion- and metastasis-related morphological characteristics include fatty (Figure 5Ce) and muscle infiltration (Figure 5Ck), bidirectional differentiation (referred to as an epithelial-mesenchymal transition characteristic; Figures 5Cf,l), and tumor emboli (Figure 5Cm) in vessels were observed in treatment group. In HCT116, some mice in treatment group had liver (Figure 5Cn) and lung metastasis (Figure 5Co). Tumor growth curves of LoVo (Figure 5Ba) and HCT116 (Figure 5Bb) inoculated groups were plotted on the basis of tumor size, measured every 2 days after tumor visualization. The data revealed that tumor growth in the treatment was significantly faster than that in control and si-treatment (Table 1).

**Table 1:** Tumor formation, metastasis, and growth among control, treatment and si-treatment cells inoculated nude mice.

**Cells**	**Group**	**n**	**Tumor formation rate (%)**	***P***	**Metastasis rate**	***P***	**Tumor growth (mm^**3**^)**	***P***
LoVo	Control	5	20.00	0.000^*^	0	NS	78.04 ± 49.30	*P* = 0.000^*^ *P*1 = 0.000^*^ *P*2 = 0.000^*^ *P*3 = 0.244
	Treatment	10	100.00		0		155.87 ± 116.14	
	Si-Treatment	15	93.33		0		102.17 ± 81.22	
HCT116	Control	5	100	NS	0	0.000^*^	145.23 ± 104.23	*P* = 0.000^*^ *P*1 = 0.000^*^ *P*2 = 0.000^*^ *P*3 = 0.722
	Treatment	10	100		30%		410.56 ± 323.76	
	Si-Treatment	10	100		0		166.85 ± 182.08	

*NS: no significance*;

* *P < 0.05 (P: difference among the three groups; P1: difference between control and treatment; P2: difference between si-treatment and treatment; P3: difference between control and si-treatment)*.

The IHC staining intensity and positive percentage of S100A4 (Figure 5Da), cathepsin B (Figure 5Db), cyclin B1 (Figure 5Dc), Annexin A2 (Figure 5Dd), S100A10 (Figure 5De), TRIM21 (Figure 5Df), and Ezrin (Figure 5Dh) gradually increased in group I, group II, group III, and group IV. No marked differences were observed among the four groups with regards to 14-3-3 ζ/δ expression (Figure 5Dg). Staining indices of S100A4, cathepsin B, cyclin B1, Annexin A2, S100A10, TRIM21, 14-3-3 ζ/δ, and Ezrin revealed significant differences (*P* = 0.000). Group II exhibited higher staining indices for S100A4 (*P* = 0.035), cathepsin B (*P* = 0.003), cyclin B1 (*P* = 0.000), Annexin A2 (*P* = 0.013), S100A10 (*P* = 0.000), TRIM21 (*P* = 0.000), and Ezrin (*P* = 0.003) than group I, except for 14-3-3 ζ/δ (*P* = 0.643), and all indices were significantly higher in group III than in group II. No significant differences were observed between group IV and group III (Table 2). S100A4, cathepsin B, cyclin B1, Annexin A2, S100A10, TRIM21, 14-3-3 ζ/δ, and Ezrin may hence participate in tumor invasion depending on the grade of human CRCs, and protein upregulation during lymph node metastasis also suggests a positive correlation with metastasis in human CRCs.

**Table 2:** Comparison of S100A4-related proteins in human CRC tissues.

**Tissues**	**Well-differentiated CRC**	**Moderately differentiated CRC**	**Poorly differentiated CRC**	**Lymph node metastasis**	***χ^2^***	***P***	***P*1**	***P*2**	***P*3**
Group	I	II	III	IV					
*n* (222)	51	55	52	64					
S100A4	2.37 ± 3.09	3.65 ± 3.38	5.34 ± 3.32	6.24 ± 2.46	42.032	0.000^*^	0.035^*^	0.013^*^	0.224
cathepsin B	2.60 ± 2.45	4.04 ± 2.39	5.28 ± 3.12	5.57 ± 2.25	35.859	0.000^*^	0.003^*^	0.041^*^	0.588
cyclin B1	2.94 ± 1.76	5.77 ± 2.40	6.81 ± 2.09	6.41 ± 2.15	71.535	0.000^*^	0.000^*^	0.030^*^	0.336
Annexin A2	2.12 ± 2.20	3.21 ± 2.39	4.39 ± 2.85	5.49 ± 2.28	46.036	0.000^*^	0.013^*^	0.041^*^	0.027^*^
S100A10	2.18 ± 2.20	4.94 ± 2.09	6.50 ± 2.41	7.34 ± 1.83	90.766	0.000^*^	0.000^*^	0.001^*^	0.085
TRIM21	2.12 ± 2.16	4.11 ± 2.26	5.67 ± 2.84	5.95 ± 2.16	63.821	0.000^*^	0.000^*^	0.006^*^	0.610
14-3-3 ζ/δ	7.52 ± 1.99	7.72 ± 1.87	8.52 ± 1.27	8.73 ± 0.87	20.551	0.000^*^	0.643	0.011^*^	0.415
Ezrin	2.24 ± 2.06	3.55 ± 2.32	4.96 ± 3.01	5.40 ± 2.17	41.859	0.000^*^	0.003^*^	0.034^*^	0.328

* *P < 0.05: statistically significant. (P: difference among the three groups; P1: difference between group I and group II; P2: difference between group II and group III; P3: difference between group III and group IV)*.

## Discussion

It is well-known that CRCs are a threat to human health worldwide because of the high morbidity associated with them, caused by the invasion and metastasis of tumor cells (1). Increasingly, studies are indicating that PGCCs with budding cells are highly correlated with malignant biological behaviors, such as tumor metastasis, recurrence, and drug-resistant (8, 10, 23). In tumor tissues, PGCCs number, single stromal PGCC, and PGCCs generating erythroid cells to form vasculogenic mimicry had been used as risk factors of CRC metastasis (8, 18, 24). The findings of this study help reveal the molecular events underlying regulation of invasion and metastasis by S100A4 in CRC PGCCs with their daughter cells.

As a metastasin, S100A4 is associated with numerous cytoskeletal proteins, such as actin, myosin, and tropomyosin, increasing the tumor progression and metastasis (19, 25–27). Cathepsin B belongs to a family of lysosomal cysteine proteases comprising disulfide-linked heavy and light chains, which can directly or indirectly degrade the extracellular matrix in tumor tissues (28). Inhibition of cathepsin B could reduce liver metastases of CRC (29). Cyclin B1 is a key cell cycle protein regulating the G2/M phase transition, contributing to the formation of PGCCs, highly correlating with the invasion and metastasis of various malignant tumors (30, 31). This study confirmed that overexpression of S100A4, cathepsin B, and cyclin B1 is associated with the increased infiltration and invasion of CRC cell lines after CoCl_2_ treatment. Increased S100A4 in the cytoplasm targeted the cytoskeleton, resulting in a change in the cell shape; the nuclear translocation of S100A4 regulated downstream targets affecting cell infiltration and invasion (32). High expression of cathepsin B promotes cell infiltration, and increase of cyclin B1 mainly locating in cytoplasm is indicative of G2/M arrest resulting in the formation of PGCCs; the daughter cells derived from PGCCs positively expressed cyclin B1 in the nucleus contributing to the high proliferation activity. PGCCs with their daughter cells are highly invasive, which was suppressed after S100A4 knockdown along with the reduced expression of cathepsin B and cyclin B1, and a decrease of cyclin B1 nuclear translocation in daughter cells. These data suggest that S100A4 may contribute to the migration and invasion of PGCCs with their daughter cells via affecting cathepsin B and cyclin B1.

Based on Co-IP of S100A4, we detected the high expression of Annexin A2, S100A10, TRIM21, 14-3-3 ζ/δ, and Ezrin in PGCCs with their daughter cells; these upregulations located primarily in the cytoplasm except for S100A10 and Ezrin were upregulated in the nucleus of daughter cells. The proteins 14-3-3 ζ/δ and Ezrin, which link cytoskeletal and membrane proteins, have also implicated in cellular movement contributing to tumorigenesis, invasion, and metastasis (33, 34). Two Annexin A2 monomers bridged by an S100A10 homodimer are known to form a multifunctional membrane-associated heterotetramer (35), which has been implicated in membrane trafficking events and membrane-cytoskeletal connection relating to cell motility and drug-resistance in human cancers (36, 37). S100A10, a small dimeric helix-loop-helix protein residing in a tight complex with Annexin A2, appeared to regulate the intracellular trafficking of a variety of membrane-resident proteins, such as cathepsin B (38). TRIM21 is an E3 ubiquitin ligase associated with autoimmune diseases (39), and the dysregulation of TRIM21 facilitates human cancer development (40, 41). This study detected a significant decrease of TRIM21 and Annexin A2 levels in PGCCs with their daughter cells after S100A4 knockdown as well as a reduced nuclear translocation of S100A10 in daughter cells, while 14-3-3 ζ/δ and Ezrin did not vary.

Studies have shown that S100A10 is ubiquitinated and degraded rapidly in the absence of Annexin A2 (42, 43). However, in this study, S100A10 expression was not affected by the decrease in Annexin A2 levels after S100A4 knockdown in PGCCs with their daughter cells, which might indicate that the decreased expression of TRIM21 directly inhibited the S100A10 ubiquitination and degradation. Followed by TRIM21 knockdown in PGCCs with their daughter cells, the expression levels of S100A4, cathepsin B, cyclin B1, and S100A10 declined, as well as a decrease of nuclear translocation of S100A4, cyclin B1, and S100A10 in daughter cells, while the others were unchanged. The mechanism by which S100A10 expression is suppressed in the presence of Annexin A2 after TRIM21 knockdown is yet to be elucidated. *In vivo* assays confirmed that the tumorigenicity of PGCCs with their daughter cells was significantly higher than the control cells, while the high tumorigenicity and metastasis dramatically declined after S100A4 knockdown. Moreover, the expression of S100A4-related proteins was positively correlated with the malignancy degree of human CRC, and maintained a high level in lymph node metastasis.

In conclusions, Annexin A2 combines with S100A10 to form a multifunctional heterotetrametric complex, which was identified as a center in the molecular network of S100A4 regulating migration and invasion in PGCCs with their daughter cells. The inhibition of Annexin A2 or S100A10 may affect the function of the complex. S100A4 and TRIM21 may regulate each other to affect cyclin B1 expression and subcellular localization contributing to the formation of PGCCs with their daughter cells, which participates in regulating the structure and function of Annexin A2/S100A10 complex, affecting downstream cathepsin B, resulting in the invasion and metastasis of PGCCs with their daughter cells. Besides, 14-3-3 ζ/δ and Ezrin may be involved in the motility and invasion of PGCCs with their daughter cells via cytoskeletal constructions with S100A4. The potential mechanisms by which S100A4 influences the invasion and metastasis of PGCCs with their daughter cells in human CRCs may provide a novel strategy for CRC therapy by targeting S100A4 molecular events. The detailed mechanisms by which S100A4 and TRIM21 regulate the Annexin A2/S100A10 complex in PGCCs and their daughter cells need to be explored further.

## Data Availability Statement

The authors declare that all data supporting the findings of this study are available within the article and its Supplementary Material or contact the corresponding author upon reasonable request.

## Ethics Statement

The animal study was approved by the Institutional Animal Care and Use Committee of Tianjin Union Medicine Center. All the human CRC tissue samples involved in our study were paraffin-embedded tissues *in vitro* obtained from the department of pathology of Tianjin Union Medical Center, which did not require the written informed consent from the patients. The use of human tissue samples was approved by the Hospital Review Board of Tianjin Union Medicine Center and the confidentiality of patient information was maintained.

## Author Contributions

SZ accessed funding, designed the study, and contributed to original manuscript writing. FF, KL, and CL collected, analyzed, and interpreted data. JD, ZW, and BL conducted methodology and formal analysis. YL and YZ gave constructive comments on the manuscript. All authors contributed to writing-review and editing and approved the manuscript before submission.

### Conflict of Interest

The authors declare that the research was conducted in the absence of any commercial or financial relationships that could be construed as a potential conflict of interest.

## Abbreviations

CRC: colorectal cancer
CoCl_2_: cobalt chloride
PGCCs: polyploid giant cancer cells
FBS: fetal bovine serum
PBS: phosphate-buffered saline
ICC: immunocytochemical
WB: western blotting
siRNAs: small interfering RNAs
PC: positive control
NC: negative control
MC: mock control
Co-IP: co-immunoprecipitation
MS: mass spectrometry
H&E: hematoxylin and eosin
IHC: immunohistochemical
TRIM21: tripartite motif-containing 21.

## References

[1] Bray F Ferlay J Soerjomataram I Siegel RL Torre LA Jemal A. Global cancer statistics 2018: GLOBOCAN estimates of incidence and mortality worldwide for 36 cancers in 185 countries. CA Cancer J Clin. (2018) 68:394–424. 10.3322/caac.21492 30207593

[2] Khan N Mukhtar H. Cancer and metastasis: prevention and treatment by green tea. Cancer Metastasis Rev. (2010) 29:435–45. 10.1007/s10555-010-9236-1 20714789 PMC3142888

[3] Siegel R Naishadham D Jemal A. Cancer statistics, 2013. CA Cancer J Clin. (2013) 63:11–30. 10.3322/caac.21166 23335087

[4] Lopez-Sanchez LM Jimenez C Valverde A Hernandez V Penarando J Martinez A. CoCl2, a mimic of hypoxia, induces formation of polyploid giant cells with stem characteristics in colon cancer. PLoS ONE. (2014) 9:e99143. 10.1371/journal.pone.0099143 24932611 PMC4059626

[5] Zhang S Mercado-Uribe I Xing Z Sun B Kuang J Liu J. Generation of cancer stem-like cells through the formation of polyploid giant cancer cells. Oncogene. (2014) 33:116–28. 10.1038/onc.2013.96 23524583 PMC3844126

[6] Fei F Zhang D Yang Z Wang S Wang X Wu Z. The number of polyploid giant cancer cells and epithelial-mesenchymal transition-related proteins are associated with invasion and metastasis in human breast cancer. J Exp Clin Cancer Res. (2015) 34:158. 10.1186/s13046-015-0277-8 26702618 PMC4690326

[7] Zhang S Zhang D Yang Z Zhang X. Tumor budding, micropapillary pattern, and polyploidy giant cancer cells in colorectal cancer: current status and future prospects. Stem Cells Int. (2016) 2016:4810734. 10.1155/2016/4810734 27843459 PMC5097820

[8] Lv H Shi Y Zhang L Zhang D Liu G Yang Z. Polyploid giant cancer cells with budding and the expression of cyclin E, S-phase kinase-associated protein 2, stathmin associated with the grading and metastasis in serous ovarian tumor. BMC Cancer. (2014) 14:576. 10.1186/1471-2407-14-576 25106448 PMC4137091

[9] Zhang L Ding P Lv H Zhang D Liu G Yang Z. Number of polyploid giant cancer cells and expression of EZH2 are associated with VM formation and tumor grade in human ovarian tumor. Biomed Res Int. (2014) 2014:903542. 10.1155/2014/903542 25025074 PMC4082869

[10] Chen J Niu N Zhang J Qi L Shen W Donkena KV. Polyploid giant cancer cells (PGCCs): the evil roots of cancer. Curr Cancer Drug Targets. (2019) 19:360–7. 10.2174/1568009618666180703154233 29968537

[11] Zhang S Mercado-Uribe I Hanash S Liu J. iTRAQ-based proteomic analysis of polyploid giant cancer cells and budding progeny cells reveals several distinct pathways for ovarian cancer development. PLoS ONE. (2013) 8:e80120. 10.1371/journal.pone.0080120 24348907 PMC3858113

[12] Watanabe Y Usuda N Tsugane S Kobayashi R Hidaka H. Calvasculin, an encoded protein from mRNA termed pEL-98, 18A2, 42A, or p9Ka, is secreted by smooth muscle cells in culture and exhibits Ca(2+)-dependent binding to 36-kDa microfibril-associated glycoprotein. J Biol Chem. (1992) 267:17136–40. 1512251

[13] Ravasi T Hsu K Goyette J Schroder K Yang Z Rahimi F. Probing the S100 protein family through genomic and functional analysis. Genomics. (2004) 84:10–22. 10.1016/j.ygeno.2004.02.002 15203200

[14] Ebralidze A Tulchinsky E Grigorian M Afanasyeva A Senin V Revazova E. Isolation and characterization of a gene specifically expressed in different metastatic cells and whose deduced gene product has a high degree of homology to a Ca2+-binding protein family. Genes Dev. (1989) 3:1086–93. 10.1101/gad.3.7.1086 2550322

[15] Davies BR Davies MP Gibbs FE Barraclough R Rudland PS. Induction of the metastatic phenotype by transfection of a benign rat mammary epithelial cell line with the gene for p9Ka, a rat calcium-binding protein, but not with the oncogene EJ-ras-1. Oncogene. (1993) 8:999–1008. 8455951

[16] Li ZH Dulyaninova NG House RP Almo SC Bresnick AR. S100A4 regulates macrophage chemotaxis. Mol Biol Cell. (2010) 21:2598–610. 10.1091/mbc.e09-07-0609 20519440 PMC2912347

[17] Dahlmann M Kobelt D Walther W Mudduluru G Stein U. S100A4 in cancer metastasis: wnt signaling-driven interventions for metastasis restriction. Cancers. (2016) 8:59. 10.3390/cancers8060059 27331819 PMC4931624

[18] Zhang D Yang X Yang Z Fei F Li S Qu J. Daughter cells and erythroid cells budding from PGCCs and their clinicopathological significances in colorectal cancer. J Cancer. (2017) 8:469–78. 10.7150/jca.17012 28261349 PMC5332899

[19] Fei F Zhang M Li B Zhao L Wang H Liu L. Formation of polyploid giant cancer cells involves in the prognostic value of neoadjuvant chemoradiation in locally advanced rectal cancer. J Oncol. (2019) 2019:2316436. 10.1155/2019/2316436 31558902 PMC6735173

[20] Liu K Lu R Zhao Q Du J Li Y Zheng M. Association and clinicopathologic significance of p38MAPK-ERK-JNK-CDC25C with polyploid giant cancer cell formation. Med Oncol. (2019) 37:6. 10.1007/s12032-019-1330-9 31734829

[21] Sun B Zhang S Zhang D Yin X Wang S Gu Y. Doxycycline influences microcirculation patterns in B16 melanoma. Exp Biol Med. (2007) 232:1300–7. 10.3181/0705-RM-145 17959842

[22] Takizawa CG Morgan DO. Control of mitosis by changes in the subcellular location of cyclin-B1-Cdk1 and Cdc25C. Curr Opin Cell Biol. (2000) 12:658–65. 10.1016/S0955-0674(00)00149-6 11063929

[23] Xuan B Ghosh D Cheney EM Clifton EM Dawson MR. Dysregulation in actin cytoskeletal organization drives increased stiffness and migratory persistence in polyploidal giant cancer Cells. Sci Rep. (2018) 8:11935. 10.1038/s41598-018-29817-5 30093656 PMC6085392

[24] Yang Z Yao H Fei F Li Y Qu J Li C. Generation of erythroid cells from polyploid giant cancer cells: re-thinking about tumor blood supply. J Cancer Res Clin Oncol. (2018) 144:617–27. 10.1007/s00432-018-2598-4 29417259 PMC11813446

[25] Fei F Qu J Zhang M Li Y Zhang S. S100A4 in cancer progression and metastasis: A systematic review. Oncotarget. (2017) 8:73219–39. 10.18632/oncotarget.18016 29069865 PMC5641208

[26] He Z Yu L Luo S Li M Li J Li Q. miR-296 inhibits the metastasis and epithelial-mesenchymal transition of colorectal cancer by targeting S100A4. BMC Cancer. (2017) 17:140. 10.1186/s12885-017-3121-z 28209128 PMC5311719

[27] Zhang J Hou S Gu J Tian T Yuan Q Jia J. S100A4 promotes colon inflammation and colitis-associated colon tumorigenesis. Oncoimmunology. (2018) 7:e1461301. 10.1080/2162402X.2018.1461301 30221056 PMC6136879

[28] Mehrotra S Wickremesekera SK Brasch HD Van Schaijik B Marsh RW Tan ST. Expression and localization of cathepsins B, D and G in cancer stem cells in liver metastasis from colon adenocarcinoma. Front Surg. (2018) 5:40. 10.3389/fsurg.2018.00040 30177970 PMC6110174

[29] Nadal C Maurel J Gascon P. Is there a genetic signature for liver metastasis in colorectal cancer? World J Gastroenterol. (2007) 13:5832–44. 10.3748/wjg.v13.i44.5832 17990349 PMC4205430

[30] Song Y Zhao C Dong L Fu M Xue L Huang Z. Overexpression of cyclin B1 in human esophageal squamous cell carcinoma cells induces tumor cell invasive growth and metastasis. Carcinogenesis. (2008) 29:307–15. 10.1093/carcin/bgm269 18048386

[31] Li W Dong Q Li L Zhang Z Cai X Pan X. Prognostic significance of claudin-1 and cyclin B1 protein expression in patients with hypopharyngeal squamous cell carcinoma. Oncol Lett. (2016) 11:2995–3002. 10.3892/ol.2016.4333 27123052 PMC4840523

[32] Tarabykina S Griffiths TR Tulchinsky E Mellon JK Bronstein IB Kriajevska M. Metastasis-associated protein S100A4: spotlight on its role in cell migration. Curr Cancer Drug Targets. (2007) 7:217–28. 10.2174/156800907780618329 17504119

[33] Pan Y Zhong LJ Zhou H Wang X Chen K Yang HP. Roles of vimentin and 14-3-3 zeta/delta in the inhibitory effects of heparin on PC-3M cell proliferation and B16-F10-luc-G5 cells metastasis. Acta Pharmacol Sin. (2012) 33:798–808. 10.1038/aps.2012.42 22669117 PMC4010382

[34] Fadiel A Choi SD Park B Kim TH Buldo-Licciardi J Ahmadi M Expression of ezrin and estrogen receptors during cervical carcinogenesis. Reprod Sci. (2017) 24:706–12. 10.1177/1933719116667222 27688241

[35] Hedhli N Falcone DJ Huang B Cesarman-Maus G Kraemer R Zhai H. The annexin A2/S100A10 system in health and disease: emerging paradigms. J Biomed Biotechnol. (2012) 2012:406273. 10.1155/2012/406273 23193360 PMC3496855

[36] Suzuki S Tanigawara Y. Forced expression of S100A10 reduces sensitivity to oxaliplatin in colorectal cancer cells. Proteome Sci. (2014) 12:26. 10.1186/1477-5956-12-26 24851084 PMC4029833

[37] Huang D Yang Y Sun J Dong X Wang J Liu H. Annexin A2-S100A10 heterotetramer is upregulated by PML/RARalpha fusion protein and promotes plasminogen-dependent fibrinolysis and matrix invasion in acute promyelocytic leukemia. Front Med. (2017) 11:410–22. 10.1007/s11684-017-0527-6 28687976

[38] Rescher U Gerke V. S100A10/p11: family, friends and functions. Pflugers Arch. (2008) 455:575–82. 10.1007/s00424-007-0313-4 17638009

[39] Rhodes DA Isenberg DA. TRIM21 and the function of antibodies inside cells. Trends Immunol. (2017) 38:916–26. 10.1016/j.it.2017.07.005 28807517

[40] Nguyen JQ Irby RB. TRIM21 is a novel regulator of Par-4 in colon and pancreatic cancer cells. Cancer Biol Ther. (2017) 18:16–25. 10.1080/15384047.2016.1252880 27830973 PMC5323013

[41] Itou J Li W Ito S Tanaka S Matsumoto Y Sato F. Sal-like 4 protein levels in breast cancer cells are post-translationally down-regulated by tripartite motif-containing 21. J Biol Chem. (2018) 293:6556–64. 10.1074/jbc.RA117.000245 29511085 PMC5925811

[42] Puisieux A Ji J Ozturk M. Annexin II up-regulates cellular levels of p11 protein by a post-translational mechanisms. Biochem J. (1996) 313:51–5. 10.1042/bj3130051 8546709 PMC1216908

[43] He KL Deora AB Xiong H Ling Q Weksler BB Niesvizky R. Endothelial cell annexin A2 regulates polyubiquitination and degradation of its binding partner S100A10/p11. J Biol Chem. (2008) 283:19192–200. 10.1074/jbc.M800100200 18434302 PMC2443646

