# Supplementary material for: Molecular Mechanisms by Which S100A4 Regulates the Migration and Invasion of PGCCs With Their Daughter Cells in Human Colorectal Cancer
Source: Front Oncol. 2020 Feb 21;10:182. doi: 10.3389/fonc.2020.00182 (PMC7047322; doi:10.3389/fonc.2020.00182)
Supplement: Supplementary file 1 [file Image_1.pdf]

Supplementary Figure legends

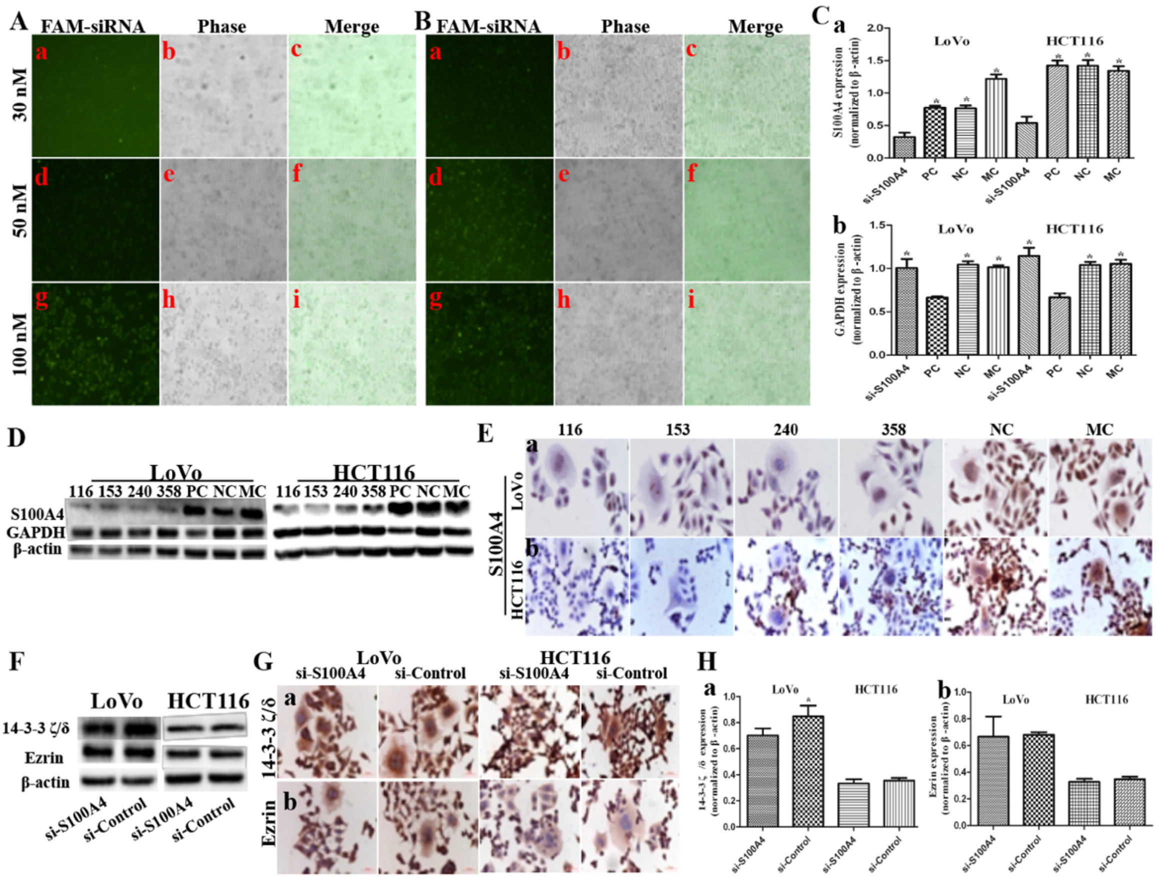

Figure S1. Determination of siRNA transfection efficiency using fluorescently labeled siRNAs (FAM-siRNA) (100×) and the interfering efficiency of S100A4 in PGCCs and their daughter cells. A. At a 10:1 proportion of siRNA (μl) and lipofectamine (pmol). (a) Cells transfected with 30 nM FAM-siRNA, viewed under fluorescent light. (b) Cells transfected with 30nM FAM-siRNA, viewed in bright field. (c) Merge image of (a) and (b). (d) Cells transfected with 50 nM FAM-siRNA, viewed under fluorescent light. (e) Cells transfected with 50nM FAM-siRNA, viewed in bright field. (f) Merge image of (d) and (e). (g) Cells transfected with 100 nM FAM-siRNA, viewed under fluorescent light. (h) Cells transfected with 100nM FAM-siRNA, viewed in bright field. (i) Merge image of (g) and (h). B. At a 20:1 proportion of siRNA (μl) and lipofectamine (pmol). (a) Cells transfected with 30 nM FAM-siRNA, viewed under fluorescent light. (b) Cells transfected with 30nM FAM-siRNA, viewed in bright field. (c) Merge image of (a) and (b). (d) Cells transfected with 50 nM FAM-siRNA, viewed under fluorescent light. (e) Cells transfected with 50nM FAM-siRNA, viewed in bright field. (f) Merge image of (d) and (e). (g) Cells transfected with 100 nM FAM-siRNA, viewed under fluorescent light. (h) Cells transfected with 100nM FAM-siRNA, viewed in bright field. (i) Merge image of (g) and (h). C. Comparison of western blot band intensities for S100A4 (a) and GAPDH (b) before and after S100A4 knockdown (values normalized to that of β-actin; $^{*}P<0.05$). D. Western blot analysis of interfering efficiency of S100A4 and GAPDH (PC). E. ICC staining of inhibition of S100A4 in LoVo (a) and HCT116 (b) cells. F. WB showed the expression of 14-3-3 ζ/δ and Ezrin before and after S100A4 knockdown. G. ICC staining of 14-3-3 ζ/δ (a) and Ezrin (b) before and after S100A4 knockdown. H. Bar graph of WB band intensities for 14-3-3 ζ/δ (a) and Ezrin (b) before and after S100A4 knockdown.

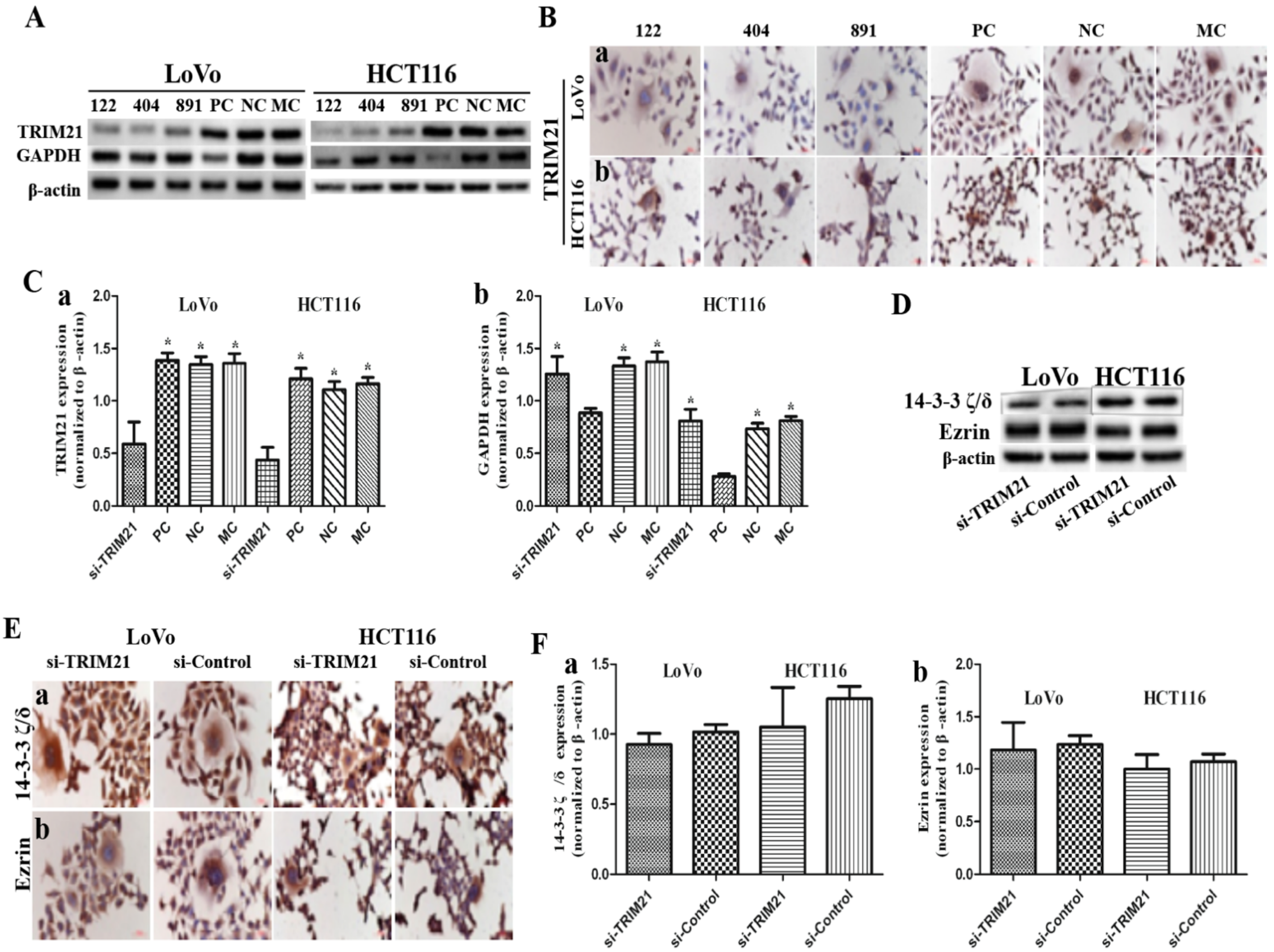

Figure S2. Determination of interfering efficiency of TRIM21 in PGCCs and their daughter cells and the expression of 14-3-3 ζ/δ and Ezrin before and after TRIM21 knockdown. A. Western blot analysis of interfering efficiency of TRIM21 and GAPDH (PC). B. ICC staining of inhibition of TRIM21 in LoVo (a) and HCT116 (b) cells. C. Comparison of western blot band intensities for TRIM21 (a) and GAPDH (b) before and after TRIM21 knockdown (values normalized to that of β-actin; $^{*}P<0.05$). D. WB showed the expression of 14-3-3 ζ/δ and Ezrin before and after TRIM21 knockdown. E. ICC staining of 14-3-3 ζ/δ (a) and Ezrin (b) before and after TRIM21 knockdown. F. Bar graph of WB band intensities for 14-3-3 ζ/δ (a) and Ezrin (b) before and after TRIM21 knockdown.

Supplementary full original blots:

Figure-1F

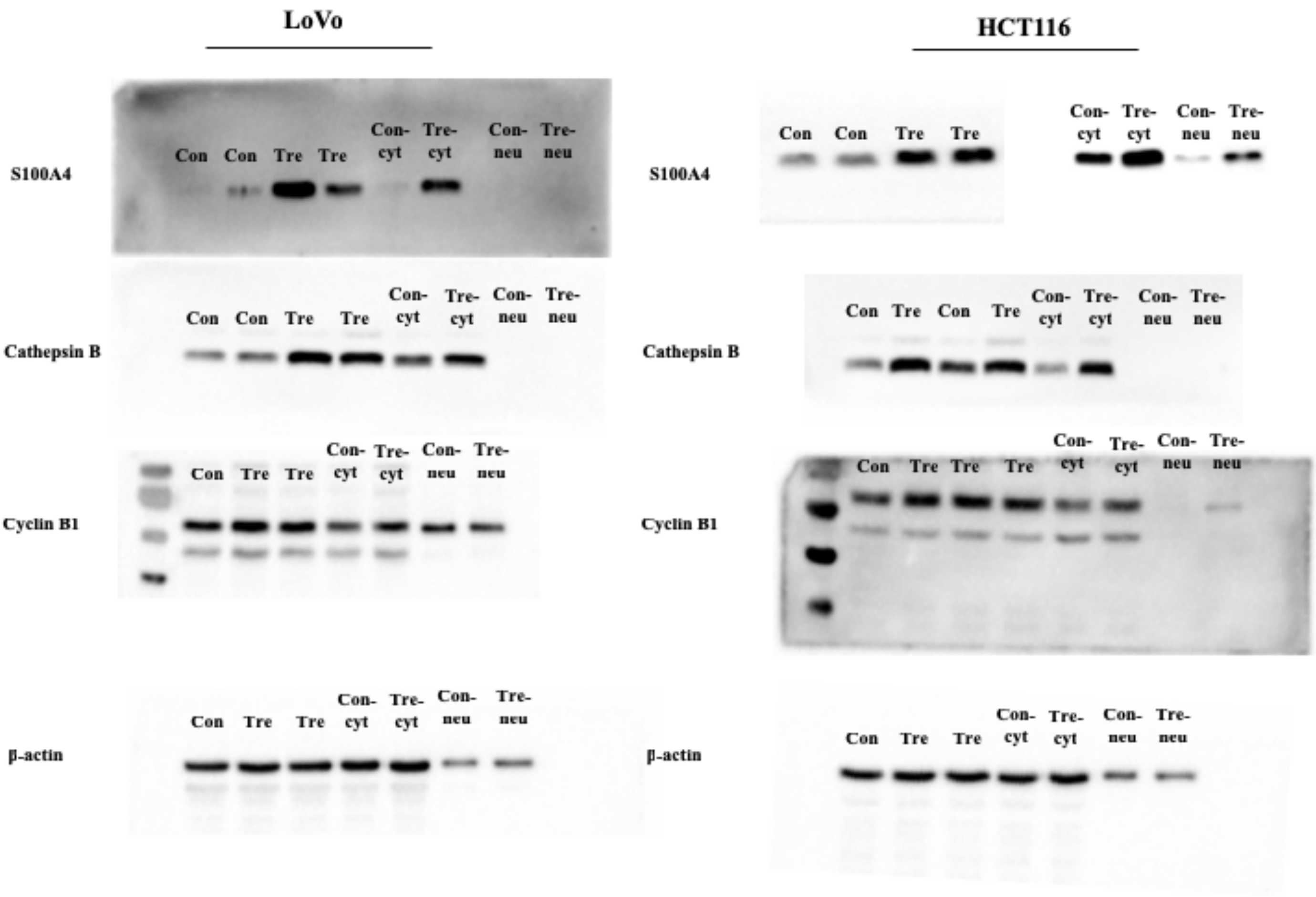

Figure-2A

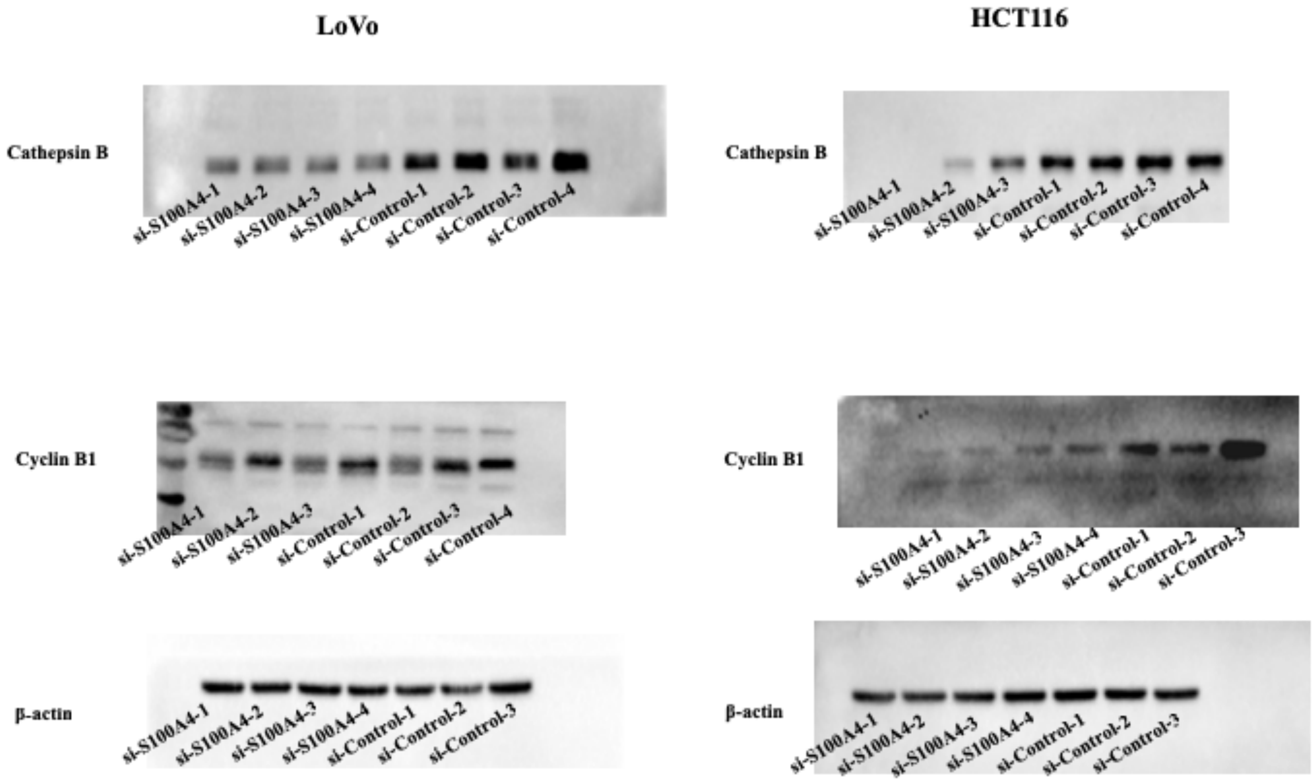
LoVo
HCT116
Cathepsin B
si-S100A4-1
si-S100A4-2
si-S100A4-3
si-S100A4-4
si-Control-1
si-Control-2
si-Control-3
si-Control-4
Cathepsin B
si-S100A4-1
si-S100A4-2
si-S100A4-3
si-Control-1
si-Control-2
si-Control-3
si-Control-4
Cyclin B1
si-S100A4-1
si-S100A4-2
si-S100A4-3
si-Control-1
si-Control-2
si-Control-3
si-Control-4
Cyclin B1
si-S100A4-1
si-S100A4-2
si-S100A4-3
si-S100A4-4
si-Control-1
si-Control-2
si-Control-3
β-actin
si-S100A4-1
si-S100A4-2
si-S100A4-3
si-S100A4-4
si-Control-1
si-Control-2
si-Control-3
β-actin
si-S100A4-1
si-S100A4-2
si-S100A4-3
si-S100A4-4
si-Control-1
si-Control-2
si-Control-3

Figure-2F

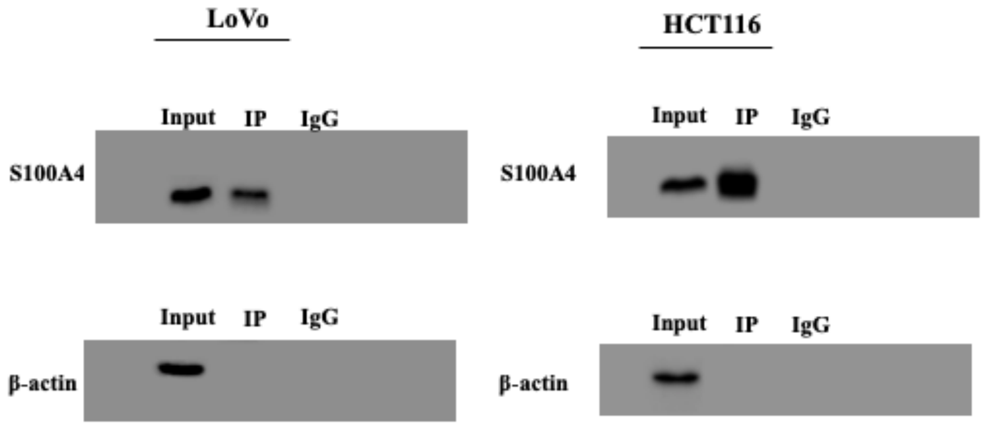
LoVo
HCT116
Input
IP
IgG
S100A4
Input
IP
IgG
S100A4
Input
IP
IgG
β-actin
Input
IP
IgG
β-actin

Figure-2G

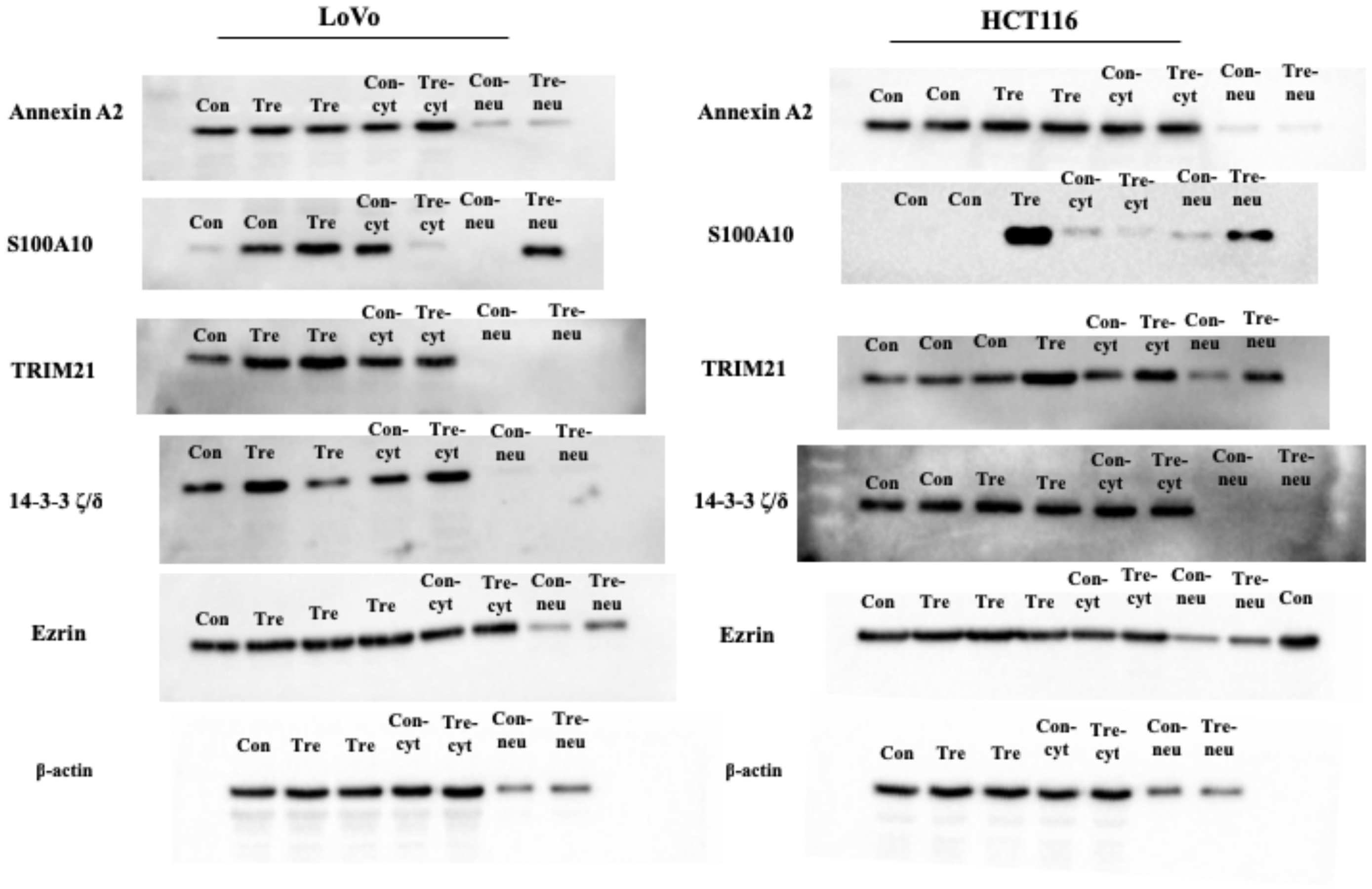
LoVo
HCT116
Annexin A2
S100A10
TRIM21
14-3-3 ζ/δ
Ezrin
β-actin
Con
Tre
Con-cyt
Tre-cyt
Con-neu
Tre-neu

Figure-3A

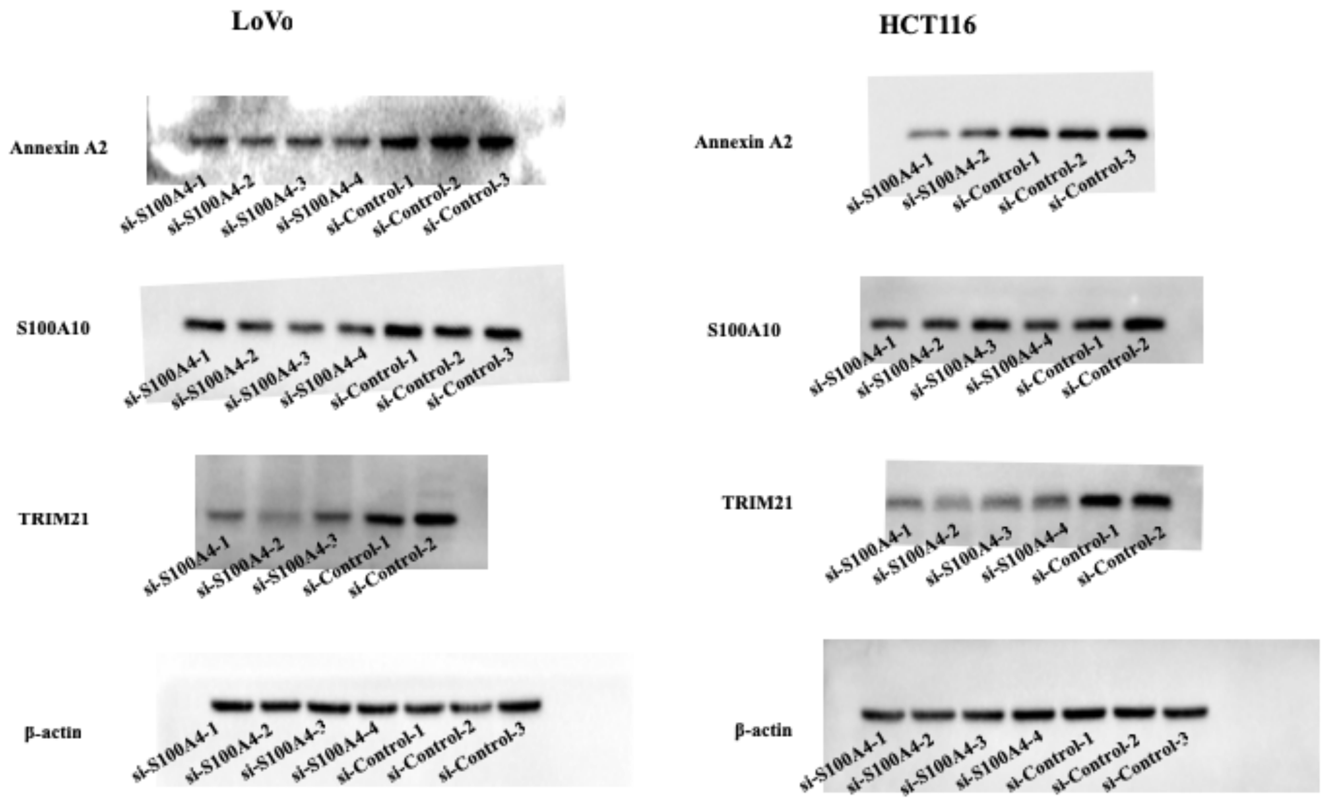
LoVo
HCT116
Annexin A2
si-S100A4-1 si-S100A4-2 si-S100A4-3 si-S100A4-4 si-Control-1 si-Control-2 si-Control-3
Annexin A2
si-S100A4-1 si-S100A4-2 si-Control-1 si-Control-2 si-Control-3
S100A10
si-S100A4-1 si-S100A4-2 si-S100A4-3 si-S100A4-4 si-Control-1 si-Control-2 si-Control-3
S100A10
si-S100A4-1 si-S100A4-2 si-S100A4-3 si-S100A4-4 si-Control-1 si-Control-2
TRIM21
si-S100A4-1 si-S100A4-2 si-S100A4-3 si-Control-1 si-Control-2
TRIM21
si-S100A4-1 si-S100A4-2 si-S100A4-3 si-S100A4-4 si-Control-1 si-Control-2
β-actin
si-S100A4-1 si-S100A4-2 si-S100A4-3 si-S100A4-4 si-Control-1 si-Control-2 si-Control-3
β-actin
si-S100A4-1 si-S100A4-2 si-S100A4-3 si-S100A4-4 si-Control-1 si-Control-2 si-Control-3

Figure-3D

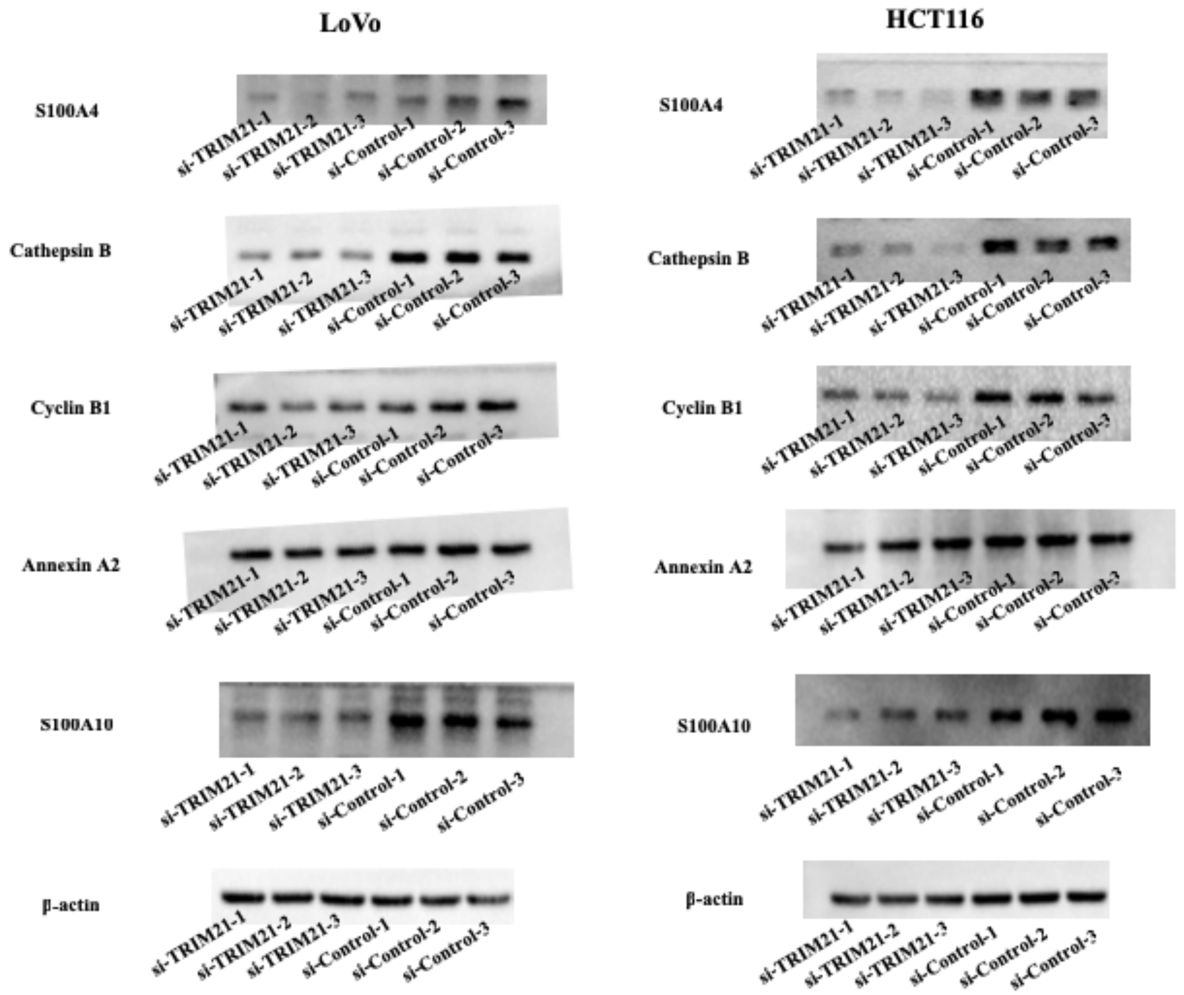
LoVo
HCT116
S100A4
Cathepsin B
Cyclin B1
Annexin A2
S100A10
β-actin
si-TRIM21-1
si-TRIM21-2
si-TRIM21-3
si-Control-1
si-Control-2
si-Control-3

Figure-S1F

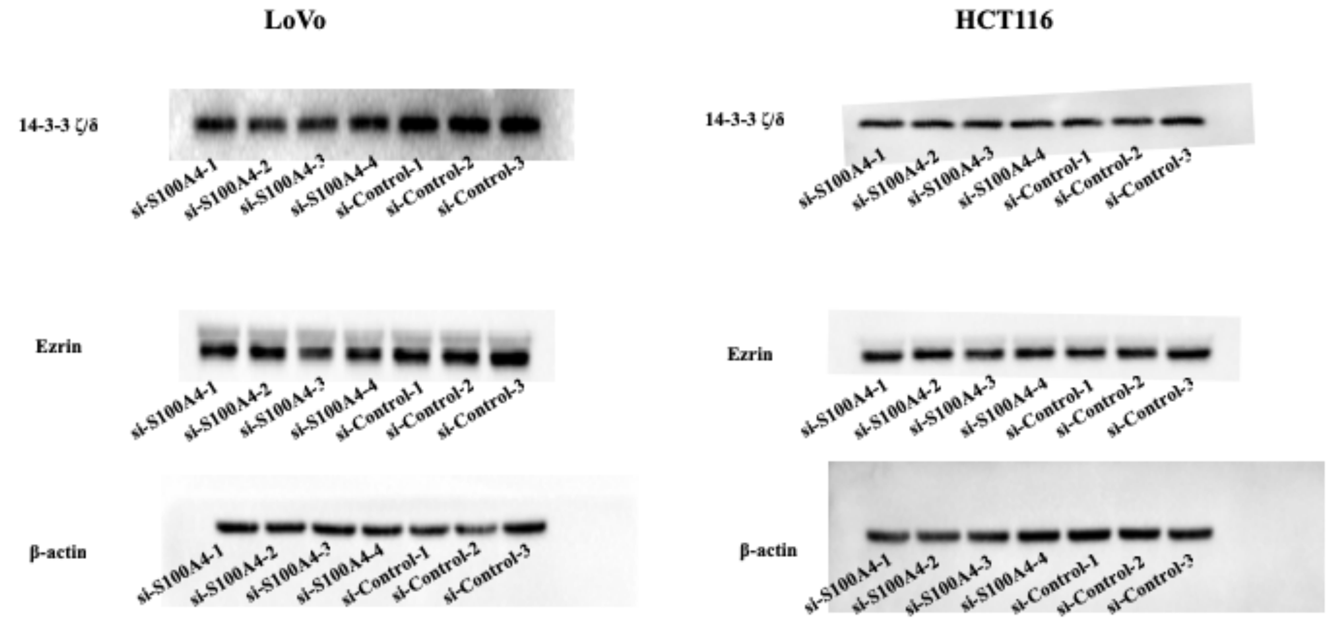
LoVo
HCT116
14-3-3 ζ/δ
si-S100A4-1
si-S100A4-2
si-S100A4-3
si-S100A4-4
si-Control-1
si-Control-2
si-Control-3
Ezrin
β-actin

Figure-S2D

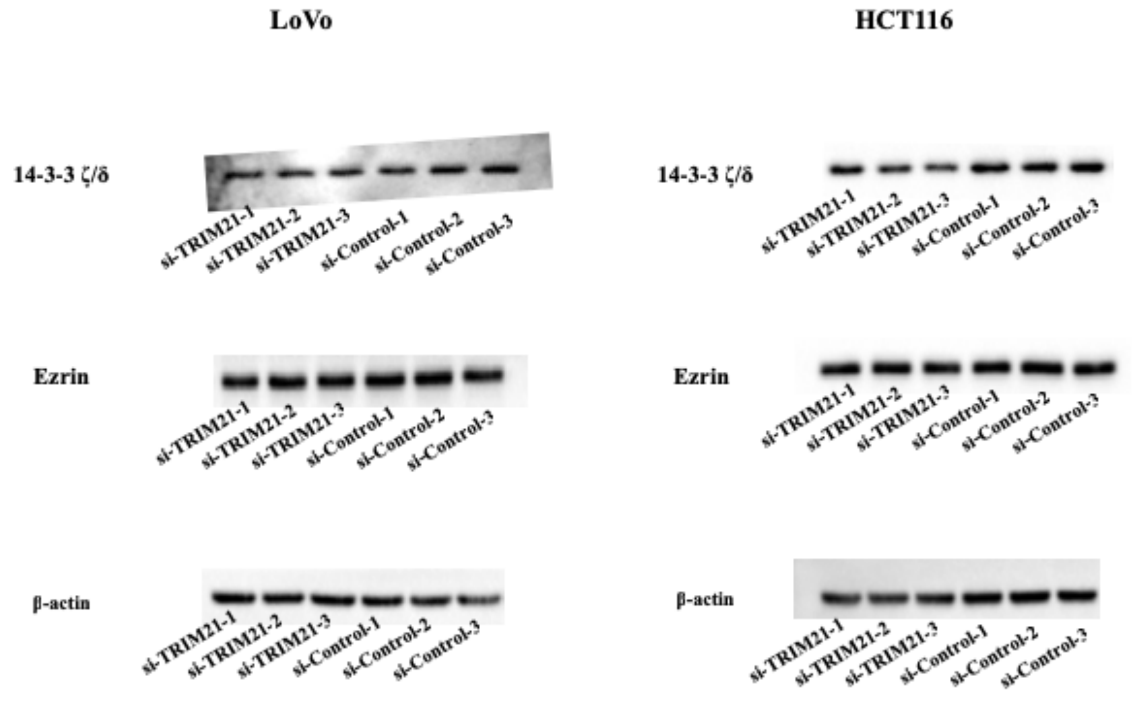
LoVo
HCT116
14-3-3 ζ/δ
Ezrin
β-actin
si-TRIM21-1
si-TRIM21-2
si-TRIM21-3
si-Control-1
si-Control-2
si-Control-3
